# EDock: blind protein–ligand docking by replica-exchange monte carlo simulation

**DOI:** 10.1186/s13321-020-00440-9

**Published:** 2020-05-27

**Authors:** Wenyi Zhang, Eric W. Bell, Minghao Yin, Yang Zhang

**Affiliations:** 1 College of Information Science and Technology, Northeast Normal University, 2555 Jingyue Street, Changchun, 130117 China; 2 Department of Computational Medicine and Bioinformatics, University of Michigan, 100 Washtenaw Avenue, Ann Arbor, MI 48109 USA; 3 Department of Biological Chemistry, University of Michigan, Ann Arbor, MI 48109-2218 USA

**Keywords:** Blind docking, REMC, Low-resolution predicted structure

## Abstract

Protein–ligand docking is an important approach for virtual screening and protein function annotation. Although many docking methods have been developed, most require a high-resolution crystal structure of the receptor and a user-specified binding site to start. This information is, however, not available for the majority of unknown proteins, including many pharmaceutically important targets. Developing blind docking methods without predefined binding sites and working with low-resolution receptor models from protein structure prediction is thus essential. In this manuscript, we propose a novel Monte Carlo based method, EDock, for blind protein–ligand docking. For a given protein, binding sites are first predicted by sequence-profile and substructure-based comparison searches with initial ligand poses generated by graph matching. Next, replica-exchange Monte Carlo (REMC) simulations are performed for ligand conformation refinement under the guidance of a physical force field coupled with binding-site distance constraints. The method was tested on two large-scale datasets containing 535 protein–ligand pairs. Without specifying binding pockets on the experimental receptor structures, EDock achieves on average a ligand RMSD of 2.03 Å, which compares favorably with state-of-the-art docking methods including DOCK6 (2.68 Å) and AutoDock Vina (3.92 Å). When starting with predicted models from I-TASSER, EDock still generates reasonable docking models, with a success rate 159% and 67% higher than DOCK6 and AutoDock Vina, respectively. Detailed data analyses show that the major advantage of EDock lies in reliable ligand binding site predictions and extensive REMC sampling, which allows for the implementation of multiple van der Waals weightings to accommodate different levels of steric clashes and cavity distortions and therefore enhances the robustness of low-resolution docking with predicted protein structures.

## Introduction

Most proteins perform their biological functions through interactions with other molecules in cells. Elucidating how proteins interact with their binding partners (or ligands) is a critical step towards understanding the function of proteins and/or designing new drugs to regulate them. In this regard, protein–ligand docking, a molecular modeling technique to predict ligand–protein binding conformations, can be used to help identify drug-like leads through virtual screening [1]. Due to the importance of docking, a number of computational approaches have been developed, with widely-used methods including GOLD [2], Glide [3], AutoDock Vina [4], DOCK [5] and others.

Many successful docking approaches are based on a hierarchical conformational searching strategy guided by various composite energy functions. For example, AutoDock Vina [4] couples an iterative search with a global optimizer, followed by the Broyden-Fletcher-Goldfarb-Shanno local optimizations guided by a knowledge-based scoring function inspired by XSCORE [6]. In DOCK6 [7], which is the latest version of the DOCK program, a ligand is disassembled into rigid segments with the largest anchor segments being oriented first to the binding sites by graph matching. The ligand positions are then sampled by growing the remaining segments till the full molecule is restored. The scoring function, which is used for pruning and minimizing the ligand conformers, contains various intra-ligand and ligand-receptor interaction terms, as well as RMSD restraint scores.

Despite the success of these docking approaches, several limitations can constrain their usefulness in practical applications. First, many of the most commonly used docking programs require the ligand binding pocket to be specified a priori by users. Although correct assignment of binding pockets can reduce the conformational search space and increase docking accuracy, the native binding pocket may be unknown to the users in many situations, especially when the ligand interaction and receptor structure are poorly characterized. Blind docking, docking a ligand without any prior knowledge of the target pocket, can be used to address these cases. Reflective of the importance of these approaches, the SAMPL (Statistical Assessment of Modeling of Proteins and Ligands) [8] challenges have been held to promote the development and predictive power of such methods. Another common limitation of the docking programs is that the methods are designed only for docking on high-resolution experimental receptor structures. Given that only ~ 150,000 out of 140 million known protein sequences in the UniProt database have solved structures in the PDB, most proteins, including many therapeutically important targets, do not have experimental structures. Although most current docking approaches succeed on docking against high-resolution experimental structures, they are highly sensitive to errors in binding pocket conformation. Since model binding pockets are often warped, as the modeling process focuses primarily on global fold accuracy rather than local binding pocket fidelity, traditional docking approaches such as DOCK6 and Vina will likely produce poses far from the native pose in an attempt to accommodate the model’s imprecise binding pocket. The accuracy of such approaches when docking against low-resolution modeled structures leaves a great deal of room for improvement. Thus, the development of docking methods applicable to low-resolution predicted protein structures is an important issue.

In this work, we propose a novel Monte Carlo simulation method, EDock, which aims at high-quality blind docking on low-resolution predicted protein structures. Starting from a 3D structure of the protein or a protein model, such as those generated by I-TASSER [9], the structure prediction algorithm used in this manuscript, the ligand binding site and initial binding pocket are detected by a sequence-profile and local structure-based comparative approach. Next, replica-exchange Monte Carlo (REMC) simulations are performed for extensive docking conformation searching and structure refinement. To carefully examine the strength and weakness of this pipeline, we tested it on two large protein–ligand sets collected from DUDE [10] and COACH [11], which demonstrate a significant advantage of EDock over other state-of-the-art docking methods. The on-line server and the standalone program of EDock, as well as all the datasets used in this study, are made freely available at https://zhanglab.ccmb.med.umich.edu/EDock/.

## Methods

**Fig. 1:**
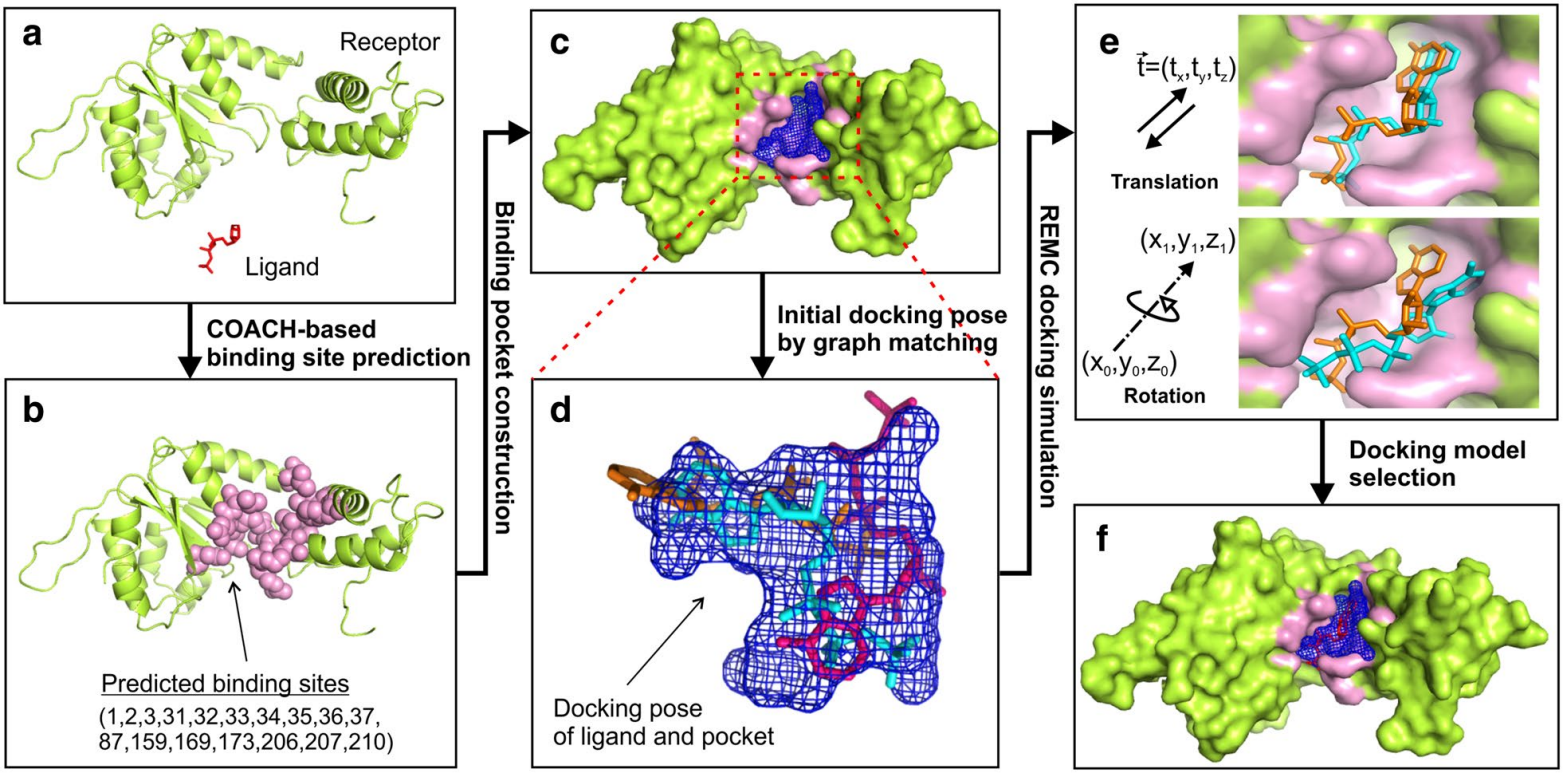
Flowchart of EDock for rigid-body blind ligand docking. **a** Input structures of protein and ligand molecules. In case a sequence is input, I-TASSER will be used to construct the structure model. **b** Profile-based binding site prediction. **c** Generation of the ligand-binding pocket. **d** Creation of initial conformations by graph matching. **e** Conformation sampling by REMC simulations. **f** Docking model selection

EDock consists of five consecutive steps: ligand-binding site prediction, binding pocket construction, initial docking pose generation, REMC docking simulation, and final model selection. A flowchart of EDock is illustrated in Fig. 1.

### Ligand binding site prediction

Starting with a protein structure, the ligand binding sites are predicted by an extension of COACH [11], a meta-server approach to binding site prediction through combining prediction results from S-SITE, TM-SITE, COFACTOR [12], FINDSITE [13] and ConCavity [14]. Here, S-SITE infers binding sites from the homologous protein templates that are detected by sequence-profile comparisons from the BioLiP library [15]. TM-SITE and COFACTOR are also comparative binding site prediction approaches but with the functional templates recognized by substructure and global-and-local based structure comparisons, respectively. FINDSITE and ConCavity are two-third-party programs, which detect binding sites using threading and structural surface cavity recognition, respectively. In EDock, the final binding site predictions are selected from results of the five predictors through a linear support vector machine consensus model. The probability of a residue to be a binding residue is calculated from individual methods, which are used as the feature vectors for the residue. The consensus classification was trained on the 400 non-redundant training proteins. Finally, all the selected binding sites are clustered based on inter-reside distances, where the coordinate center of the binding residues in the largest cluster is used as the initial binding pocket center.

### Binding pocket construction from binding site prediction

To compute the binding pocket, a cubic box with an edge length of 20 Å is created, which has its origin located at the center of the predicted binding sites. This box is represented by a set of evenly distributed grid points with a grid space of 2 Å. The inner shape of the binding pocket is then obtained through negative imaging by removing the following grid points: (1) points located less than 2.5 Å or more than 4.5 Å from any receptor atoms; (2) singleton grid points with less than 3 adjacent grid points; (3) solvent-exposed points as described in Additional file 1: Figure S1; (4) points whose distance to the box center is larger than the ligand radius, which is defined as the distance between the ligand center and the farthest ligand atom. The remaining grid points obtained after these filters are used to represent the docking pocket (Fig. 1c).

In case that predicted structures are used, the local binding pocket of the receptor can be severely distorted. Therefore, EDock will slightly reduce the cutoff in filter (1) and collect grid points located within 2–4.5 Å of the receptor atoms. If less than 10 pocket grid points remain, the subsequent filters will be skipped to ensure the number of grid points is enough to create reasonable initial conformations.

### Initial docking poses constructed by graph matching

**Fig. 2:**
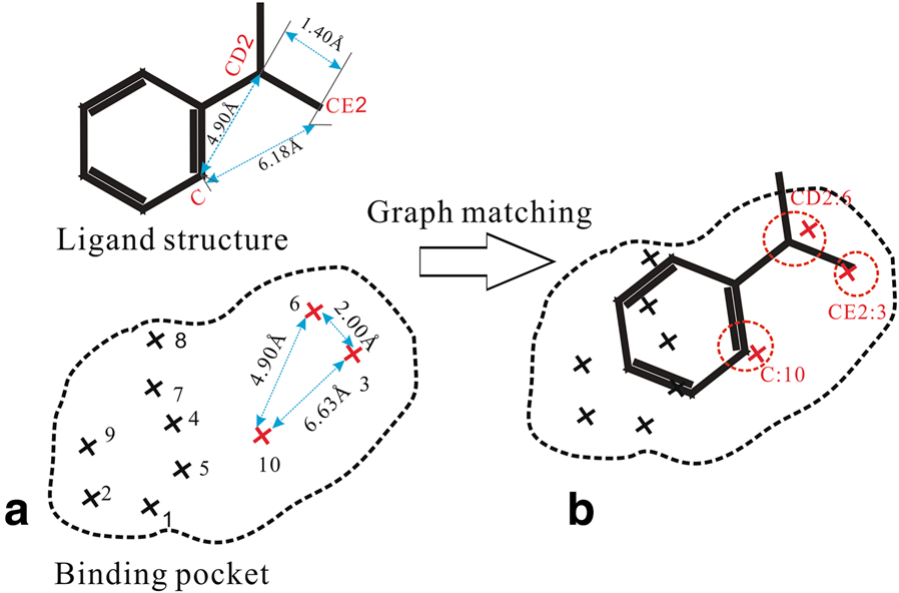
An illustration of the graph matching method in EDock. **b** The ligand structure (in sticks) is to be matched with the binding pocket, which is represented by a set of indexed crosses. **b** Three illustrative nodes are highlighted in circles, including node1 (C:10), node2 (CD2:6), and node3 (CE2:3). The intra-ligand distances ($d_{C - CD2}^{ligand} = 4.90$, $d_{C - CE2}^{ligand} = 6.18$, and $d_{CE2 - CD2}^{ligand} = 1.40$ Å) are close to the intra-pocket distances ($d_{10 - 6}^{pocket} = 4.90$, $d_{10 - 3}^{pocket} = 6.63$, and $d_{3 - 6}^{pocket} = 2.00$ Å), which results in three edges (dashed lines) being added between the nodes following Eq. (1). The three-edged nodes thus form a graph clique. Based on the atom–atom correspondence specified by the graph clique, the initial docking poses are generated by superimposing the ligand structure with the grid points of the binding pocket using the Kabsch RMSD rotation matrix [16]

Construction of the initial ligand-docking conformations with appropriate orientation and diversity is important for improving the efficiency of the subsequent docking sampling simulations. EDock uses a modified graph matching algorithm extended from Ewing et al. [5] to generate the initial docking conformations, as illustrated in Fig. 2.

During the graph matching procedure, a “node” of the graph is defined as a pair of ligand atom and grid point of the binding pocket. An “edge” is then added between two nodes (*i* and *j*) if the atom-to-atom distance within the ligand is close to the corresponding point-to-point distance in the pocket grid, i.e.,

1 $$\left| {d_{i,j}^{ligand} - d_{i,j}^{pocket} } \right| < 0.25\times n_{edge}$$

where $n_{edge}$ is the order number of accepted edges (e.g., $n_{edge} = 1$ for the first edge and 2 for the second edge etc.). From the matched graph, a clique, also known as a complete subgraph, is defined as a set of nodes in which every pair of nodes is edged. Based on the alignments specified by the cliques, the initial docking poses can be generated by superimposing the ligand structure with the corresponding grid points of the binding pocket using the Kabsch RMSD rotation matrix [16].

### Ligand–protein rigid-body docking by Monte Carlo simulation

#### REMC protocol

The ligand–protein structure refinement in EDock is performed through rigid-body docking Monte Carlo (MC) simulations. In the classic Metropolis MC protocol [17], a Markov chain of docking conformations is created by randomly moving the initial ligand conformation. At each step, the modified conformation is accepted with the probability of

2 $$p_{local} = min\left\{ {1,exp\left( { - \frac{\Delta E}{kT}} \right)} \right\}$$

where $\Delta E$ is the energy difference between new and old conformations, and $kT$ is the temperature parameter. Since the acceptance rate is exponentially reduced with the energy difference at a given temperature, the simulation can be easily trapped at a local minimum. To improve the sampling efficiency, EDock implements the REMC protocol [18], in which *N* replicas of the docking system are sampled in parallel (Additional file 1: Figure S2). The temperature of the *i*th replica is set by.

3 $$T_{i} = T_{min} \times \left( {\frac{{T_{max} }}{{T_{min} }}} \right)^{{\frac{i - 1}{N - 1}}}$$

where *T*_*min*_ = 1 and *T*_*max*_ = 60 kcal mol^−1^ R^−1^ are the lowest and highest temperatures for the first and last replicas, respectively. In every 200 local MC movements, a global swap movement between two contiguous replicas (*i* and *j*) is attempted with the acceptance probability of.

4 $$p_{global} = min\left\{ {1,exp\left( {\left( {E_{j} - E_{i} } \right)\left( {\frac{1}{{kT_{j} }} - \frac{1}{{kT_{i} }}} \right)} \right)} \right\}$$

where *E*_*j*_ and *E*_*i*_ are the energies of the *j*th and *i*th replicas. This movement can help to drive the simulation of low-temperature replicas out of local energy basins by swapping conformations with high-temperature replicas.

For each target, 5 independent REMC simulation runs are performed, where *N *= 20 (or 40) replicas are implemented in each run for experimental (or I-TASSER predicted) receptor structures. Each replica in the REMC starts with a different conformation, obtained from the graph matching process. Here, only the conformations whose energy (see Eq. 5) is < 1E + 6 kcal/mol are selected, as this energy cutoff can result in approximately 200–300 conformations with reasonable ligand RMSD (Additional file 1: Table S1), which approximately agrees with the number of 200 (= 5 × 40) replicas. Meanwhile, considering the distortion of binding pockets in predicted receptor models, 20 top-ranked conformations are selected and randomly inversed by 180° to get another 20 conformations. In case that the number of initial conformations (*M*) is < 5*N*, 5*N*-*M* new initial conformations will be generated by randomly rotating the *M* existing conformations to enhance the conformational diversity. Additional file 1: Figure S3 presents a typical example of energy trajectories from 20 different replicas from the rabbit phosphoglucose isomerase complexed with sorbitol-6-phosphate (COACH ID: 1xtbA_BS01_S6P), where Additional file 1: Table S2 lists the average acceptance rates of the swap movements; these data suggest that the current parameter setting can make reasonable replica-exchanges in the EDock simulations.

#### Energy force field

The force field in the EDock simulation contains five energy terms from ligand-receptor van der Waals, Coulombic electrostatic interactions, distance restraints to the predicted binding pocket points, distance profiles from template ligands and intra-ligand van der Waals:

5 $$E = \mathop \sum \limits_{i \in protein} \mathop \sum \limits_{j \in ligand} \left[ {w_{1} \left( {\frac{{A_{ij} }}{{d_{ij}^{12} }} - \frac{{B_{ij} }}{{d_{ij}^{6} }}} \right) + w_{2} \frac{{q_{i} q_{j} }}{{4d_{ij} }}} \right] + w_{3} \mathop \sum \limits_{k = 1}^{{n_{bind} }} d_{k} + w_{4} \mathop \sum \limits_{i \in BS} \mathop \sum \limits_{j \in ligand} \frac{1}{2} \times \frac{{\left( {d_{ij} - \mu_{ij} } \right)^{2} }}{{\sigma_{ij}^{2} }} + w_{5} \mathop \sum \limits_{i,j \in ligand, i \ne j} \left( {\frac{{C_{ij} }}{{s_{ij}^{12} }} - \frac{{D_{ij} }}{{S_{ij}^{6} }}} \right)$$

where $d_{ij}$ is the distance between the *i*th atom in the protein and the *j*th atom in the ligand. $A_{ij} = \varepsilon R^{12}$ and $B_{ij} = 2\varepsilon R^{6}$ are repulsion and attraction parameters, where $R = r_{i} + r_{j}$ and $\varepsilon = \sqrt {\varepsilon_{i} \varepsilon_{j} }$ are related to van der Waals radii and well depths, respectively, the values of which are taken from AMBER99 [19] and listed in Additional file 1: Table S3. Out of the many widely used physical force fields used in biomolecule simulations, such as AMBER, CHARMM, and OPLS, we chose the AMBER force field, which is shown to be useful for ligand–protein docking by previous works [7]. The “DockPrep” module of Chimera is used for adding the hydrogen atoms and partial charges for both receptor and ligand. In the Coulombic interaction term, $q_{i}$ and $q_{j}$ are the atom charges, which are determined using Amber’s Antechamber module [20]. The third term in Eq. (5) is to constrain the ligand molecule towards the binding pocket predicted by the second step of EDock (Fig. 1c), where $n_{bind}$ is the number of grid points in the pocket, and $d_{k}$ is the minimum distance between ligand and the *k*th binding pocket point.

**Fig. 3:**
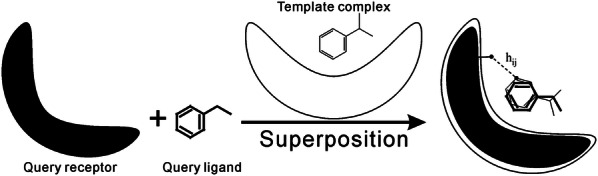
The ligand-receptor atomic distance profile heuristic. To derive the distance profile, query receptor and ligand structures are separately matched with the template-ligand complexes, where the receptor structure match is copied from the alignment generated by the first step of EDock, and the ligand structure match is made by LS-align [29]. The average distance $\mu_{ij}$ is then calculated by $\mu_{ij} = \left( {1/N} \right)\mathop \sum \nolimits_{t = 1}^{N} h_{ij} \left( t \right)$, where $h_{ij} \left( t \right)$ is the distance between *i*th atom of the query receptor and the ligand atom (at *t*th template) that corresponds to the *j*th atom of the query ligand, and $N$ is the number of template complexes which have the ligand aligned with the *j*th atom of the query ligand in the LS-align alignment. In EDock, we only consider template ligands whose LS-score is > 0.7 with the query ligand. The variance is calculated as $\sigma_{ij}^{2} = \left( {1/N} \right)\mathop \sum \nolimits_{t = 1}^{N} (h_{ij} \left( t \right) - \mu_{ij} )^{2}$

The fourth term in Eq. (5) is to constrain the distances between ligand and receptor atoms based on the original template structures of ligand-receptor complexes detected in the first step of EDock (Fig. 1b). Here, *BS* is defined as all atoms on the binding sites of the receptor, $\mu_{ij}$ is the mean reference distance calculated from template complex structures, with $\sigma_{ij}^{2}$ being the standard deviation of these distances (Fig. 3).

For targets with a high-resolution experimental receptor structure, only the van der Waals potential and Coulombic electrostatic interactions are used with equal weights, i.e., $w_{1} = w_{2} = 1$, and $w_{3} = w_{4} = 0$. In the case that low-resolution receptor structures are predicted from I-TASSER, which often involves steric clashes, a set of five different van der Waals weights (i.e., $w_{1} = 0.001, 0.004, 0.02, 0.1, 1$) are used separately in the five different simulations to accommodate different levels of ligand–protein clashes, where $w_{2} = 1$, $w_{3} = 1 - w_{1}$ and $w_{4} = 0.01$ in all of the simulations. In the Additional file 1: Table S4, we compare the performance of only one van der Waals weight and different composite sets of the weights and found that our current implementation of weights is optimal. These parameters were determined on an independent training dataset by minimizing the average ligand RMSD of the final models. Additional file 1: Table S5 shows the docking result at different box sizes and REMC swap numbers on 180 predicted structure models from the COACH dataset. Here, the box size was set as 20 Å as the average RMSD of the initial ligand pose by graph matching is minimized at this size (7.56 Å). The REMC swap number is set to 200, because with this value, we obtain the best result (7.10 Å) using a van der Waals weight equal to 1 and only one REMC simulation run.

#### Monte carlo movements

The conformational movements in the REMC simulations involve rigid-body translations and rotations of the ligand molecule (Fig. 1e). At each MC step, the ligand is first translated along the vector $$\overset{\lower0.5em\hbox{$\smash{\scriptscriptstyle\rightharpoonup}$}} {t} = \left( {t_{x} ,t_{y} ,t_{z} } \right)$$, where $$t_{x}$$, $$t_{y}$$, and $$t_{z}$$ are random numbers generated in the range of [− 0.2, 0.2]. The translated ligand is then rotated around a randomly oriented axis by a random angle in [− 3°, 3°]. To create a unit vector as the rotation axis, the starting point of the vector is set at the ligand center $$\left( {x_{0} ,y_{0} ,z_{0} } \right)$$ and the end point $$\left( {x_{1} ,y_{1} ,z_{1} } \right)$$ is randomly sampled on a unit sphere centered at the ligand center by $$x_{1} = x_{0} + \sin \varphi \cos \theta$$, $$y_{1} = y_{o} + \sin \varphi { \sin }\theta$$, and $$z_{1} = z_{0} + \cos \varphi$$ (see Additional file 1: Figure S4), where the spherical coordinate of the end point $$\left( {{{\uptheta }},\varphi } \right)$$ are calculated by $${{\uptheta }} = 2\pi {\text{r}}_{1}$$, $$\varphi = \cos^{ - 1} \left( {2r_{2} - 1} \right)$$ with $$r_{1}$$ and $$r_{2}$$ being random numbers in [0,1]. This procedure can ensure that the rotation vector is randomly oriented and evenly distributed in the rotational space, which is essential for ergodic MC sampling.

### Flexible docking

EDock also implements flexible docking. The intra-ligand van der Waals is represented in the simulation energy by the fifth term in Eq. (5). It is formulated the same as the van der Waals between receptor and ligand atoms, where $s_{ij}$ is the distance between the *i*th and *j*th atom in the ligand, and $C_{ij} = \varepsilon R^{12}$ and $D_{ij} = 2\varepsilon R^{6}$ are repulsion and attraction parameters related to the ligand van der Waals radii and well depths. The weight of this intra-ligand VDW term is equal to 1 for all simulations. For flexible movement, all rotatable bonds are determined according to which bonds are single bonds in the “BOND” information table of the MOL2 file. After the rigid body conformational translation and rotation of the whole molecule, one rotatable bond is randomly selected as the rotation axis. All atoms at the end of this rotatable bond are rotated by a random angle sampled within [− 180°,180°]. Before running the docking REMC simulation, the intra-ligand VDW energy term will be used to justify the input ligand conformation. If it is larger than 100, the single bonds of the ligand will be randomly rotated by 500 Monte Carlo simulation steps to obtain a reasonable initial conformation; otherwise, this step will be skipped. In the flexible docking REMC simulation, 40 replicas of the docking system are sampled with 200 global swap operations.

### Ranking and selection of docking conformations

For each REMC run, the docking decoys are collected from four replicas of the lowest temperatures, with each decoy picked up in every 20 MC movements. Thus, EDock generates in total 20,000 decoy conformations (= 5 × 4 × 100 × 200/20) for each target, as EDock runs 100 swaps each after 200 local MC movements. EDock provides two protocols for the final model selection: the first is to select models with the highest XSCORE, which is an empirical scoring function proposed to evaluate the binding affinity of a given protein–ligand complex structure [6]. We chose to use XSCORE instead of the EDock simulation energy for model selection, because previous observations have shown that the simulation energy is usually insensitive (or less sensitive than a third-party energy function) to the quality of decoy conformations generated based on the minimization of the simulation energy (e.g. see Fig. 3 of [21]). The second protocol is based on a decoy clustering algorithm extended from SPICKER (Additional file 1: Figure S5), where models with the highest multiplicity, which generally correspond to those of the lowest free-energy [22], are selected.

As a validation experiment, in Additional file 1: Table S6 we list a comparison of models selected by three different protocols using the EDock energy, XSCORE, and SPICKER, where the cutoff parameters in each protocol have been optimized using the same training dataset mentioned above. The result shows that XSCORE performs the best when experimental receptor structures are used, while SPICKER clustering outperforms others when predicted receptor models are used. This is intuitively understandable because the former dataset involves receptor structures with higher accuracy, and the atomic potential of XSCORE helps properly consider subtle ligand–protein docking interactions. In the latter cases, where I-TASSER models are used (which have generally lower accuracy and various local distortions), model selection from the highest frequency of occurrence is more robust than the atomic scoring function of XSCORE. Therefore, the default EDock program uses XSCORE and SPICKER, for decoys generated based on experimental and predicted receptor models, respectively.

### Control methods

As a control, we compare EDock with two widely used docking programs: AutoDock Vina [4] and DOCK6 [7]. In all our benchmarks, a rigid-body docking experiment is performed starting from the crystal ligand conformation. Unlike the blind docking program EDock, both AutoDock Vina and DOCK6 require user-specified binding sites. For fair comparisons, we ran the two control programs using the same binding site center as predicted at the first step of EDock. For AutoDock Vina, a box is defined to restrict the conformational sampling space, where the coordinate center of the EDock-predicted binding residues is set as the box center, with a box size of 15 Å in all dimensions. To ensure rigid-body docking, we set the parameter “inactivate_all_torsions” as “true” to restrain the ligand’s conformational flexibility. For DOCK6, the target receptor and ligand were prepared by UCSF Chimera, as recommended by the DOCK6 user manual, where the parameter “flexible_ligand” is set as “no” to ensure that the ligand remains rigid in docking. All other parameters in the programs use default values.

EDock is also compared with BSP-SLIM, a ligand docking program previously developed in our lab [23]. Although both BSP-SLIM and EDock are designed for blind docking, the two approaches are fundamentally different in nearly every critical step of the process. First, BSP-SLIM identifies the initial binding pocket using global structure alignment by TM-align [24], while EDock detects binding pockets by substructure and sequence profile comparisons based on an algorithm extended from COACH, which has shown to have a significantly higher accuracy than TM-align for binding-site identification [11]. Second, BSP-SLIM uses OMEGA [25] to create ligand conformations, which are then superposed onto putative binding pockets to generate final models by negative image matching. One major issue in BSP-SLIM is that the ligand conformers are pre-created and are blind to specific receptor conformations. Also, although the negative image matching is fast, it cannot sufficiently explore the complex conformational space of ligand–protein interactions. To address these issues, EDock obtains initial docking poses by a graph matching procedure that can account for specific ligand shapes and binding pocket conformations. An extensive docking conformational search is then performed by REMC simulations. Third, the energy function of BSP-SLIM relies mainly on ligand and pocket shape matching, while EDock combines multiple physics and knowledge-based terms as described in Eq. (5). Finally, BSP-SLIM selects models based on the same chemical and shape matching score from the negative imaging, while EDock selects models from a combination of XSCORE and SPICKER clustering as described in Sect. “Flexible docking”. To ensure rigid-body docking in BSP-SLIM, we set the parameter “-maxconfs” as 1 and “-includeInput” as “true” in our benchmarks.

### Evaluation metric of docking experiments

The docking performance is evaluated mainly by two metrics. The first is the root-mean-square deviation (RMSD) of the predicted ligand conformation relative to the native structure:

6 $$RMSD = \sqrt {\frac{1}{N}\mathop \sum \limits_{i = 1}^{N} \left[ {\left( {x_{i}^{p} - x_{i}^{e} } \right)^{2} + \left( {y_{i}^{p} - y_{i}^{e} } \right)^{2} + \left( {z_{i}^{p} - z_{i}^{e} } \right)^{2} } \right]}$$

where $\left( {x_{i}^{p} ,y_{i}^{p} ,z_{i}^{p} } \right)$ and $\left( {x_{i}^{e} ,y_{i}^{e} ,z_{i}^{e} } \right)$ are, respectively, the coordinates of *i*th heavy atom in the predicted model and experimental structure of the ligand. The second metric is the distance between the geometric centers of the predicted and experimental structures of the ligand.

Both RMSD and center distance can be directly computed from the coordinates of the ligands, if the receptor is from the native structure and is kept unchanged during simulations. In case that the receptor structure is created from protein structure prediction (or from the native but with its orientation changed in the final output), the receptor model will be first superimposed on the target receptor structure by TM-score [26]. The RMSD and center distance are then calculated after the ligand model is superimposed onto the receptor structures based on the same TM-score rotation matrix.

Here, it is noted that Eq. (6) based on the default atom order can result in artificially high RMSD values for ligands with symmetric structures (such as a benzene ring). We use the DockRMSD program, which was designed to identify the minimum RMSD values by a quick graph isomorphism searching algorithm [27], to evaluate the symmetry-corrected RMSD between the docking pose and native ligand conformation.

## Results

### Datasets

Two datasets are used for benchmarking the EDock method. The first is DUDE [10], which contains 102 targets with a diverse family distribution, including 26 kinases, 15 proteases, 11 nuclear receptors, 5 GPCRs, 2 ion channels, 2 cytochrome P450s, 36 other enzymes, and 5 miscellaneous proteins. For each target in DUDE, only the active compound is used in the docking tests, while decoy compounds are skipped as they do not possess a native pose to which the predicted conformation can be compared. The second dataset is taken from COACH [11], which contains 500 non-redundant proteins that harbor 812 ligands (410 natural ligand, 238 drug-like ligand and 164 metal ions). Since EDock is designed for protein ligand docking, we discard targets from this dataset possessing metal ions and large ligands with > 50 heavy atoms and > 20 rotatable bonds, which results in a final count of 433 targets. While DUDE is a widely used dataset for virtual screening and contains a wide range of protein types, the ligand types and rotatable bonds in the COACH dataset are more diverse (Additional file 1: Figure S6). Since the performance of blind docking approaches relies on both ligand binding site prediction and receptor structural accuracy, the adoption of the two datasets can provide complementary information in the testing results of the docking methods. The datasets are downloadable at https://zhanglab.ccmb.med.umich.edu/EDock/.

### Constrained ligand docking on holo-protein structures

EDock constructs ligand docking models by refining the conformations from graph matching. To examine the efficiency and the necessity of the Monte Carlo refinement process, we first tested the ligand docking approach on the easiest cases, which use experimental holo-protein receptor structures with binding sites derived from the center of each experimental ligand position. This simplification helps to rule out the impact of incorrect binding site and receptor structure prediction in the experiment.

Table 1 lists a summary of EDock performance on both the DUDE and COACH datasets. It is shown that the graph matching algorithm can generate reasonable initial conformations with an average RMSD of 4.17 (or 3.90) Å relative to the experimental structure in the DUDE (or COACH) dataset. The REMC simulations have significantly improved the ligand docking models, with the average RMSD reduced by 2.89 and 1.99 Å on the DUDE and COACH proteins, respectively. In fact, this average RMSD decrease may be underestimated because EDock failed to perform the docking on a few cases from each dataset, resulting in an RMSD > 8 Å for these cases, which in turn, increases the average RMSD values. If we consider the median RMSD, the REMC improves the initial docking conformation by nearly 3 Å for both datasets. Accordingly, EDock achieves an RMSD < 1 Å in 80.2% (or 66.7%) of the cases, while the initial graph matching does so only in 5.9% (or 8.2%) of cases in the DUDE (or COACH) dataset. The *p*-value of the average RMSD difference, using a paired Student’s t-test, is 8.1E−15 and 1.7E−39 for the DUDE and COACH datasets, respectively, which indicates that the improvements provided by the REMC simulation are statistically significant in both datasets.

**Table 1:** Summary of docking results on holo-protein structures with known ligand binding sites

Datasets	Methods	Ligand RMSD (Å)		Center distance (Å)		Ave RMSD < 1.0 Å
		Ave	Med	Ave	Med	
DUDE (101)	Initial	4.17	3.72	1.97	1.55	7
	EDock	*1.28*	*0.36*	0.74	*0.26*	*81*
	Vina	1.38	0.52	*0.67*	0.35	80
	DOCK6	1.41	0.53	0.90	0.32	81
COACH (429)	Initial	3.90	3.13	1.61	1.26	35
	EDock	*1.91*	*0.48*	*0.99*	*0.35*	*286*
	Vina	2.72	0.78	1.36	0.56	228
	DOCK6	2.72	1.46	1.14	0.77	171

Since 4 (and 1) of 535 test targets failed in Vina (and DOCK6), only 530 targets (101 from DUDE and 429 from COACH), on which all programs have a result, are shown. “Initial” refers to initial poses from graph matching with the highest XSCORE; “Ave” and “Med” represents the average and median values, respectively. The best performance is highlighted in italic font in each category

Interestingly, the EDock models have a slightly higher RMSD in the COACH dataset than that in the DUDE dataset, although the RMSD of the initial conformations in the former is lower. This is probably because binding pockets of the COACH dataset are generally larger and easier to predict, resulting in better initial poses, while the ligands in DUDE have less structural variation, and therefore, their conformations are easier to correct through the REMC simulations. To examine this hypothesis, we present in Additional file 1: Figure S7 the histogram distribution of the number of binding-pocket grid points in the DUDE and COACH datasets. It is shown that the proteins in the COACH dataset have on average a much larger pocket size (42.86 vs 18.66) with much higher variability (standard deviation = 24.81 vs 9.89) than the proteins in DUDE; this data is consistent with the above observations on EDock performance.

As a control, we also list the performance of two widely used docking methods, AutoDock Vina and DOCK6. As it is observed, EDock outperforms both methods in all the test values, except for the DUDE dataset in which AutoDock Vina has a slightly lower center distance between model and native due to several outliers in the EDock models. However, the median center distance is lower in EDock than Vina in the same category. Overall, the average RMSD of EDock is 1.79 Å for the 530 (= 101 + 429) test targets, which is 0.67 Å lower than AutoDock Vina and 0.68 Å than DOCK6; the *p*-value from a paired Student’s t-test is 5.5E−07 and 4.6E−08, respectively, showing that the differences between EDock and the control methods are statistically significant.

**Fig. 4:**
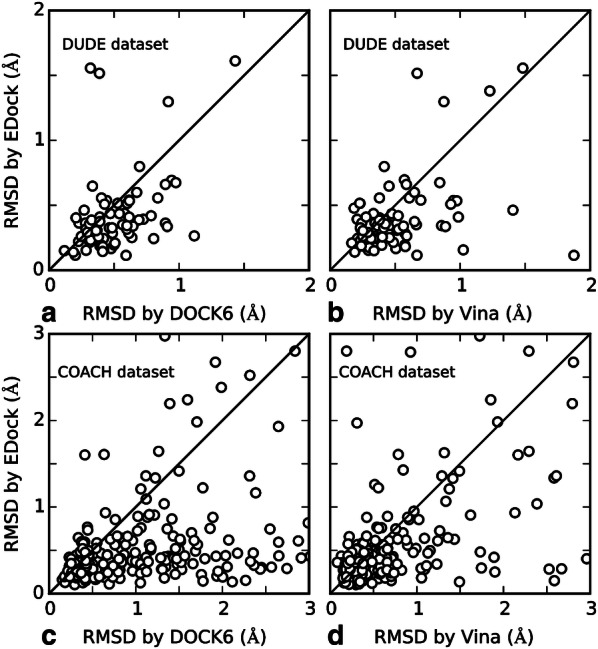
RMSD of ligand models by different methods on the holo-protein structures with known ligand binding sites. **a** EDock vs DOCK6 on DUDE dataset; **b** EDock vs AutoDock Vina on DUDE dataset; **c** EDock vs DOCK6 on COACH dataset; **d** EDock vs AutoDock Vina on COACH dataset

In Fig. 4, we present a head-to-head comparison of EDock with the two control methods on the 530 common targets in terms of ligand RMSDs. Overall, EDock has a lower RMSD than AutoDock Vina (or DOCK6) in 341 (or 373) cases, while AutoDock Vina (or DOCK6) does so in 189 (or 157) cases. The average heavy atoms and rotatable bonds are 26.28 (27.49) and 8.89 (9.18) for the cases which EDock is better than AutoDock Vina (or DOCK6), while the cases that AutoDock Vina (or DOCK6) outperforms EDock have similar averages at 26.53 (23.73) and 8.53 (7.78), respectively. Therefore, the superiority of EDock is not strongly correlated with either of these factors. When counting the number of good models with an RMSD < 1 Å, EDock has 367 cases with a good model, while AutoDock Vina and DOCK6 do so in 308 and 252 cases, respectively. These data demonstrate again the advantage of EDock in ligand docking on easy protein targets.

### Blind docking with predicted binding pockets

#### EDock generates reasonable docking models when binding site distance error is < 8 Å

In contrast to many approaches which require pre-assigned binding locations, EDock is designed to perform blind docking with initial ligand-binding sites generated from sequence-profile and substructure-based searching (Sect. “Ligand binding site prediction”). To evaluate the quality of the binding site (BS) prediction, we define BS error as the distance between the center of the predicted binding site and the center of the binding pocket in the experimental structure.

**Fig. 5:**
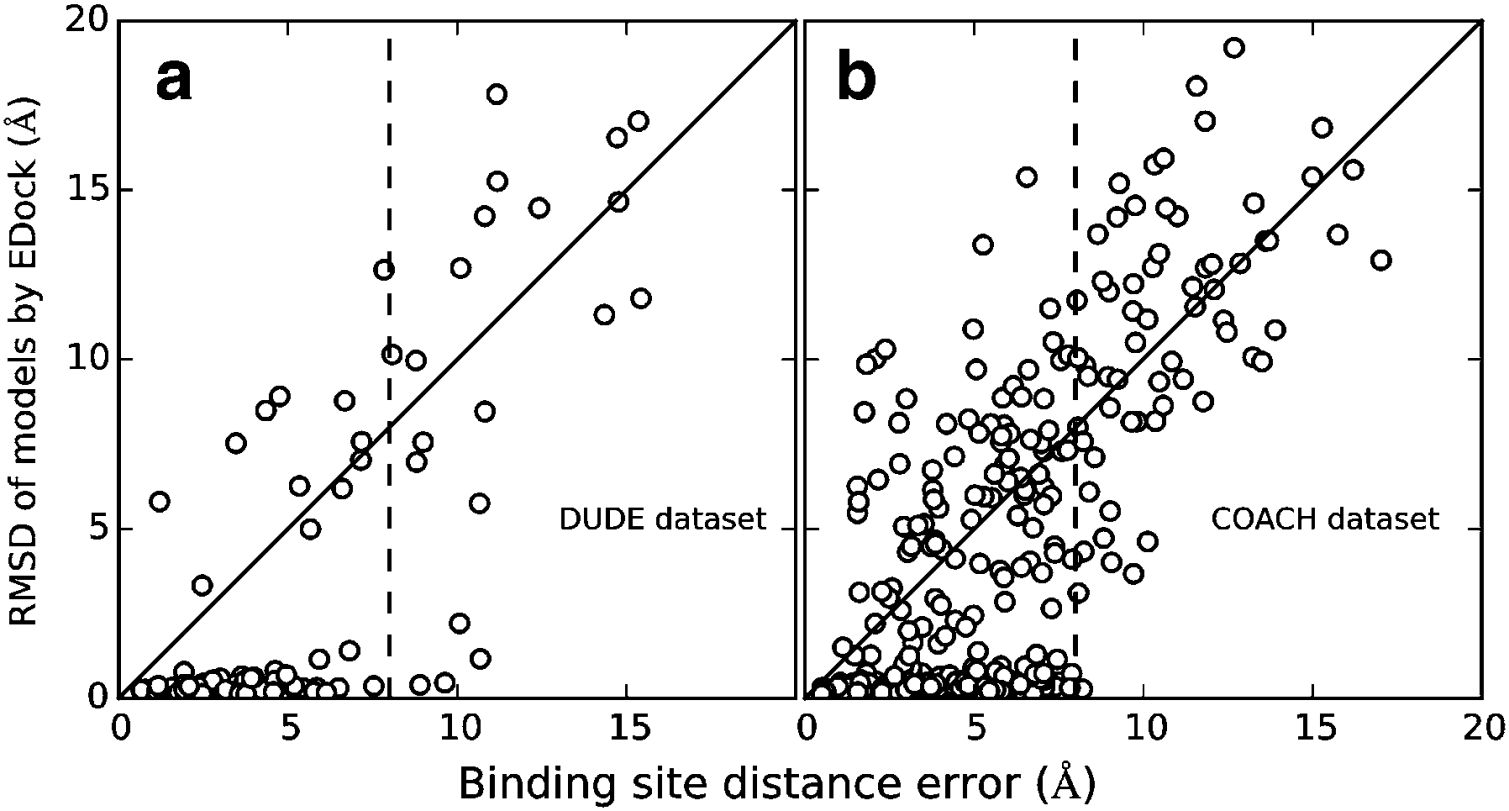
RMSD of the top ranked docking model by EDock versus the binding site distance error by COACH on the DUDE (**a**) and COACH (**b**) datasets

Figure 5 presents the dependence of the RMSD of the final models by EDock on the BS errors in both datasets. From a qualitative view of the data points, most of the targets tend to have a reasonable docking model if the BS error is below 8 Å, and vice versa (see the vertical dashed-line in the figure). In fact, the average RMSD of the docking models for the targets with BS error < 8 Å is much lower than that with BS error > 8 Å (2.17 vs 18.20 Å), which corresponds to a significant *p*-value of 6.45E−30 from an unpaired Student’s t-test. Therefore, we consider that the prediction of binding sites is acceptable if the distance error is below 8 Å. Overall, there are 76 and 315 targets whose BS error is < 8 Å for the DUDE and COACH datasets, respectively. For these targets, EDock achieves successful docking with a ligand RMSD < 2 Å in 64 and 221 cases in the two datasets, respectively, which corresponds to an overall 73% success rate.

**Fig. 6:**
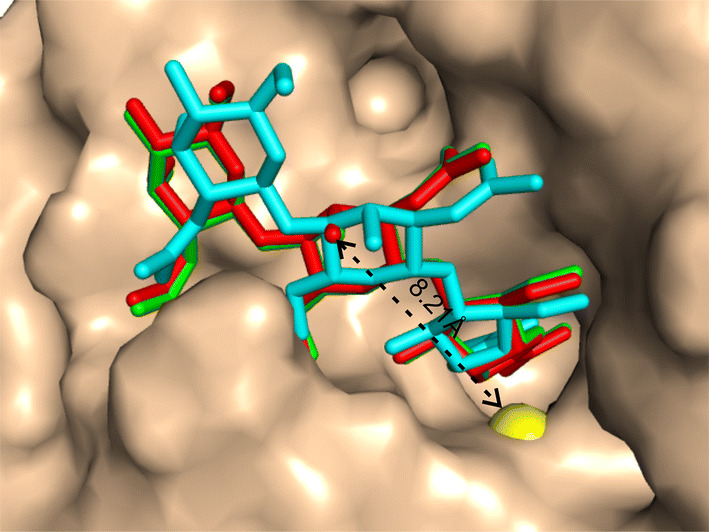
Example of successful docking on 1lloA_BS01_UUU. The center of the binding site prediction (yellow) is 8.21 Å away from the center of the native ligand (red), where graph matching detects a pose (cyan) with an RMSD of 2.12 Å. EDock constructs the final model (green) with an RMSD of 0.27 Å through REMC refinement

Even with incorrect BS predictions with a BS error > 8 Å, EDock still manages to create correct ligand-docking models with an RMSD < 2 Å for several cases. Figure 6 presents an example from the hevamine A complexed with allosamidin (COACH ID: 1lloA_BS01_UUU). For this target, the initial binding pocket prediction has a BS error of 8.21 Å, where graph matching detects multiple initial conformations with the best having an RMSD = 2.12 Å. After the REMC docking refinement, EDock creates the first model of the highest XSCORE with an RMSD = 0.27 Å to the native. This success can be mainly attributed to the extensive docking simulations which creates enough near-native conformations, where the XSCORE picks up one of the best decoys from the docking ensemble. This example also demonstrates the ability of the extended graph matching algorithm to identify acceptable initial docking pose, where EDock was able to draw the initial ligand conformation closer to the native, even without correctly predicted binding sites.

#### EDock outperforms control methods with predicted BS

In Table 2, we list a summary of the EDock models for the 391 cases in which COACH generates acceptable binding site predictions with a BS error < 8 Å. It achieves an average RMSD of 1.45 Å for the DUDE dataset and 2.17 Å for the COACH dataset, which are largely comparable to the results based on the native binding pocket (see Table 1). Here, despite the use of the BS filter, the average BS error is quite high (= 3.90 Å), which results in a high ligand RMSD of the initial docking pose (= 4.65 Å). The high accuracy of the final docking model highlights the ability of the REMC simulation of EDock to refine docking conformations when faced with low accuracy BS predictions.

**Table 2:** Summary of docking results on the targets with binding site error < 8 Å

Datasets	Methods	RMSD (Å)		Center distance (Å)		Ave RMSD < 2.0 Å
		Ave	Med	Ave	Med	
DUDE (76)	EDock	*1.45*	*0.33*	*0.88*	*0.23*	*64*
	DOCK6	2.03	0.54	1.17	0.37	57
	Vina	3.92	2.73	2.05	1.13	36
	Vina_blind_	5.15	1.15	3.50	0.92	40
COACH (315)	EDock	*2.17*	*0.43*	*1.21*	*0.33*	*221*
	DOCK6	2.84	0.90	1.56	0.51	190
	Vina	3.92	2.96	1.92	1.10	139
	Vina_blind_	7.37	6.07	5.65	2.83	101

Since 4 (and 8) test targets failed in Vina (and DOCK6), only 391 targets (76 from DUDE and 315 from COACH), on which all programs have a result, are shown. “Ave” and “Med” represent the average and median values respectively. “EDock”, “DOCK6” and “Vina” represent the results starting with the same binding sites predicted by COACH, while “Vina_blind_” refers to the blind docking result of AutoDock Vina with searching the whole receptor space. The best performance is highlighted in italic font in each category

As a control, Table 2 also lists the results from AutoDock Vina and DOCK6. The result shows that the models from EDock have a lower RMSD and center distance than those from both DOCK6 and AutoDock Vina. The *p*-value from a paired Student’s t-test is 5.95E−06 between EDock and DOCK6 and 6.48E−21 between EDock and AutoDock Vina, indicating the differences are statistically significant.

**Fig. 7:**
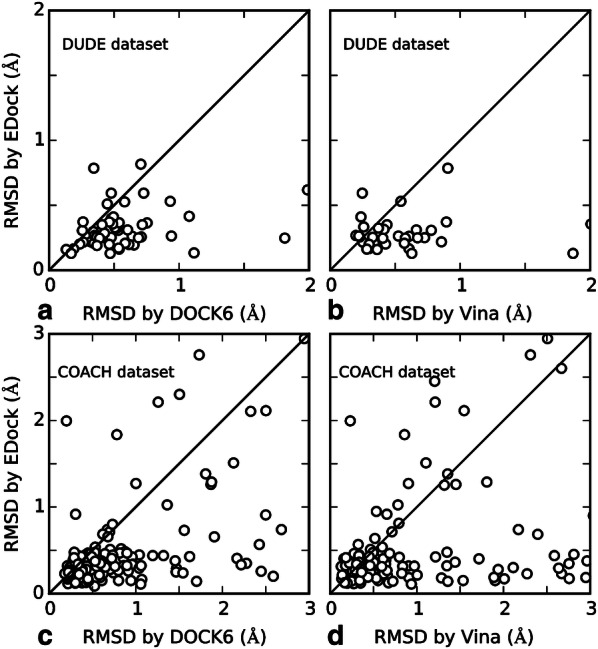
RMSD comparison of the docking models on the targets with a BS distance error below 8 Å. (**a**, **b**) DUDE dataset; (**c**, **d**) COACH dataset

In Fig. 7, we present a scatterplot comparison of EDock and the two control methods on the 391 common targets in terms of the RMSD of the top model ranked by XSCORE. Overall, there are 285 and 284 cases in which EDock created a model with a lower RMSD than Vina and DOCK6, while Vina and DOCK6 outperforms EDock only in 106 and 107 cases, respectively. If we consider a model of a ligand with an RMSD < 2 Å as successful, EDock succeeds in 285 cases, while DOCK6 and AutoDock Vina do so in 247 and 175 cases, respectively (Fig. 7).

Here, since AutoDock Vina has an option to specify the search space of docking, as a test of the ability of Vina to perform blind docking we ran the AutoDock Vina program using the whole receptor as its search space. As shown in Table 2, the models generated by Vina blind docking (represented as “Vina_blind_”) have a much higher RMSD (6.94 Å) than those generated by the program starting with the EDock predicted binding pockets (“Vina”, 3.92 Å). The number of successful cases with RMSD < 2 Å by Vina_blind_ (141) is also lower than that of Vina (175). These data suggest that the EDock binding site predictions can also be used to assist other blind docking programs.

In addition to EDock, BSP-SLIM [23] is another blind docking program which derives binding pockets from global structure alignments by TM-align. Additional file 1: Table S7 (upper panel) lists a comparison of the blind docking results by EDock and BSP-SLIM on the experimental receptor structures. Since BSP-SLIM failed on some targets, the table only lists the results for the 248 test targets for which BSP-SLIM could generate a final model. Since no BS error filter is used here, the average RMSD of the EDock models (6.49 Å) is considerably higher than that in Table 2 (1.45 Å) due to a few high-RMSD outliers for which the binding pocket prediction was completely wrong. However, the median RMSD, which is a more meaningful measure here, is equal to 1.16 Å for EDock, which is 5.2 Å lower than that of BSP-SLIM (6.36 Å), showing the significant advantage of EDock over BSP-SLIM. In addition to the different binding pocket prediction strategies, the different performance in Additional file 1: Table S7 can be mainly attributed to the extensive REMC refinement simulations by EDock, compared to the fast but simplified negative image superposition performed by BSP-SLIM.

### Blind docking on predicted protein structures

As most proteins in the UniProt database do not have experimental structures solved in the PDB, it is important to examine the ability of blind docking methods on low-resolution structures derived from protein structure prediction. This represents the most challenging case since the receptor structures are typically modeled in apo-form, where the local binding pocket conformation can be severely distorted, in addition to other possible errors in the global fold.

#### Overall results

In Table 3, we present a summary of the ligand docking results of EDock based on the receptor models predicted by I-TASSER [9]. Here, when running I-TASSER, all homologous templates with a sequence identity > 30% to the query have been excluded. As a result, the average TM-scores of the receptor models are 0.812 and 0.808, respectively, for the DUDE and COACH datasets, where there are 99 and 417 out of 102 and 433 cases in the datasets with a TM-score > 0.5. A more detailed TM-score distribution is displayed in Additional file 1: Figure S8. These results demonstrate that I-TASSER can create models of correct fold for most protein receptors even without using close homologous templates. Nevertheless, there are still a considerable number of cases where I-TASSER failed to generate correct folds. Due to these I-TASSER modeling errors, the accuracy of binding site predictions is also lower than when experimental receptor structures were used. Since docking programs will fail in most cases if the receptor structure or binding site prediction is wrong, Table 3 only focuses on the cases when the BS prediction error below 8 Å and the TM-score is larger than 0.8, in order to provide a meaningful test of the docking methods. Also, there are 6 (and 34) targets for which AutoDock Vina (and DOCK6) failed to generate a final model, due to the distortion of the local pocket structures; these cases are also skipped in Table 3.

**Table 3:** Summary of docking results on 237 targets that have receptor models from I-TASSER with TM-score > 0.8 and binding site error < 8 Å

Dataset	Method	RMSD (Å)		Center distance (Å)		Ave RMSD < 5.0 Å
		Ave	Med	Ave	Med	
DUDE (57)	EDock	*5.47*	*5.79*	*2.95*	*2.85*	*24*
	DOCK6	7.02	7.09	4.03	3.54	10
	Vina	6.91	6.96	3.41	3.16	9
COACH (180)	EDock	*4.62*	*4.28*	*2.70*	*2.11*	*100*
	DOCK6	7.05	6.89	4.43	4.24	40
	Vina	6.18	6.25	3.53	3.31	59

“Ave” and “Med” represent the average and median values respectively. The best performance is highlighted in italic font in each category

The data shows that the overall docking performance of EDock is significantly impacted by low-resolution receptor structure prediction. The average RMSD is nearly three times larger than that achieved by using the experimental holo-receptor structures, as compared to the data in Table 2. Nevertheless, EDock still outperforms DOCK6 and AutoDock Vina, with average ligand RMSDs reduced by 1.55 (2.43) and 1.44 (1.56) Å, respectively, in the DUDE (COACH) dataset. The overall improvement on the combined dataset is 2.22 and 1.53 Å relative to DOCK6 and AutoDock Vina, which corresponds to a *p*-value of 8.40E−20 and 3.20E−13, respectively, by a paired Student’s t-test, showing that the difference is statistically significant when using EDock-predicted binding pockets. If the EDock-predicted binding pocket is not used, the ligand RMSD of Vina_blind_ increases to 10.48 Å, which is significantly higher than both EDock (*p*-value = 1.90E−29) and AutoDock Vina with COACH prediction (*p*-value = 8.28E−19). Additional file 1: Table S7 (lower panel) also lists the blind docking results of EDock compared to BSP-SLIM on the 248 common targets, where the average (and median) RMSD of EDock is 1.3 (1.2) Å lower than that of BSP-SLIM; this data is consistent with the blind docking results using experimental receptor structures.

**Fig. 8:**
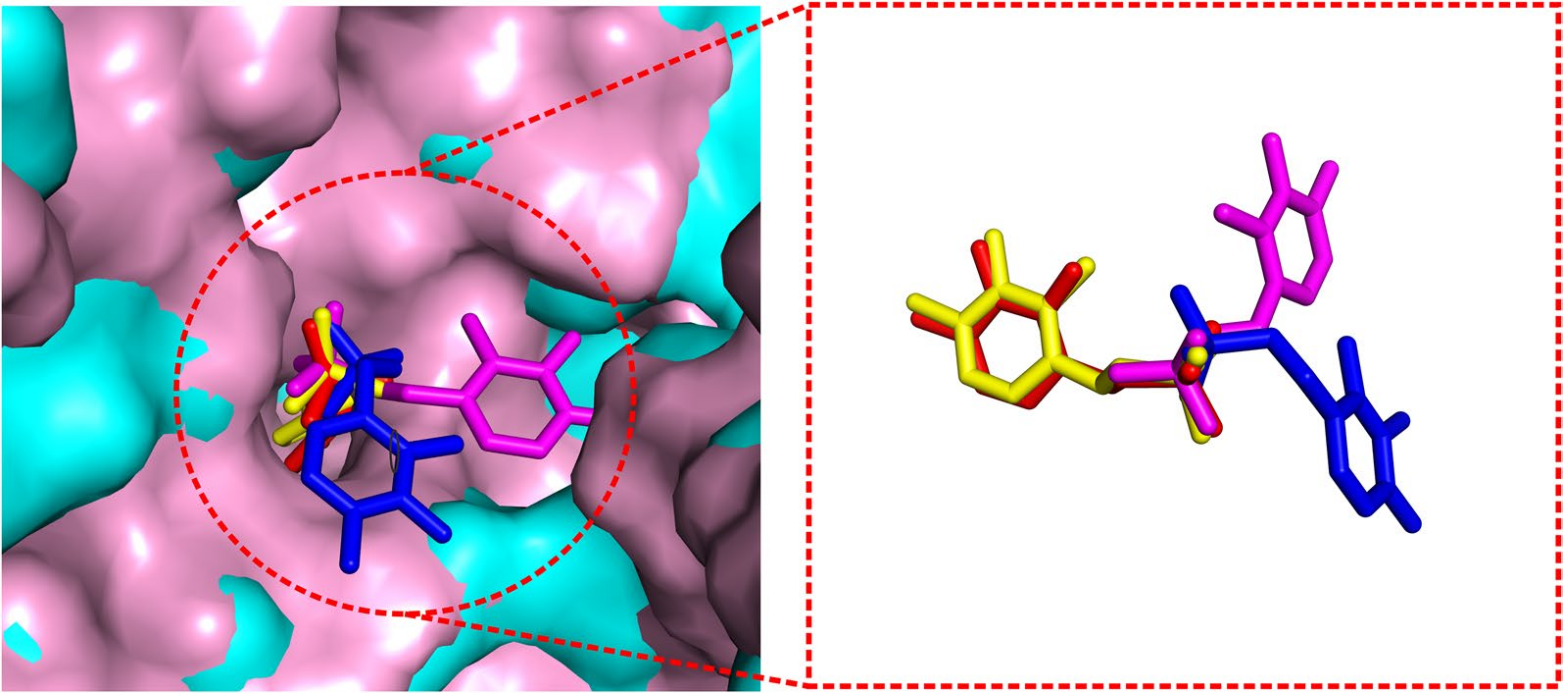
The illustration of ligand docking from 3du4A_BS01_PLP based on I-TASSER predicted models. The ligand poses by EDock (yellow), AutoDock Vina (magenta), and DOCK6 (blue) have an RMSD of 0.52, 6.46, and 8.16 Å from native (red), respectively. The background shows the superposition of the I-TASSER model (pink) on the native structure (cyan) of the receptor structure with a TM-score = 0.956

Additional file 1: Figure S9 presents a head-to-head comparison of EDock with the two control methods (DOCK6 and Vina) in terms of ligand RMSD based on I-TASSER receptor models with EDock-predicted binding pockets. Overall, EDock has a lower RMSD than Vina (DOCK6) in 162 (184) out of the 237 cases, while Vina (DOCK6) does so in 75 (53) cases, which confirms the superior performance of EDock. Figure 8 shows a representative example from the 7, 8-diaminopelargonic acid synthase bound with 7-keto-8-aminopelargonic acid (COACH ID: 3du4A_BS01_PLP), where EDock achieves a reasonable docking RMSD (0.52 Å), compared to 6.46 Å by AutoDock Vina and 8.16 Å by DOCK6 respectively.

#### Ability to accommodate local clashes inside binding pocket

One obstacle in low-resolution docking on predicted receptor structures is the distortion of the binding pocket, which can result in steric clashes between ligand and receptor atoms. Sometimes, the initial conformations obtained from graph matching can be incorrect or even oriented in the opposite direction from the native ligand due to distortion and the reduced size of the binding cavity. Since the degrees of pocket distortion and steric clashes are different for different targets, EDock adopts a strategy specifically designed for the cases with predicted receptor structures. First, it lowers the grid point cutoff to increase the variation of binding pocket creations (Sect. “Binding pocket construction from binding site prediction”). Second, EDock selects the top 20 docking poses and flips the orientation of each pose to enhance the diversity of initial binding conformations. Third, it couples the energy force field with the binding distance restraints in Eq. (5) and adopts a composite set of van der Waals weights (i.e., $w_{1}$ = 0.001, 0.004, 0.02, 0.1, and 1), which correspond to the variable binding constraint weights of ($w_{3}$ = 0.999, 0.996, 0.98, 0.9, 0) in five parallel REMC simulation runs so that the simulations can accommodate different levels of pocket distortion and steric clashes.

**Fig. 9:**
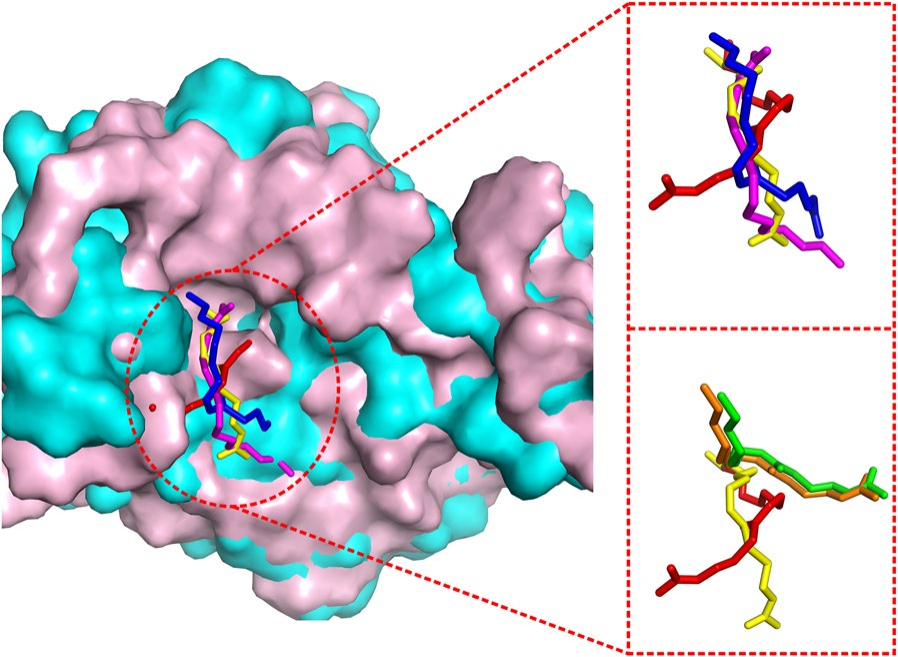
The docking results under different van der Waals potential and binding pocket distance restraint weights for 1mkaA_BS02_DAC. The I-TASSER model (cyan) has an extra steric bump inside the binding cavity, compared to the native receptor structure (pink) in the background. The upper-right inset shows the docking poses by EDock (yellow), AutoDock Vina (magenta), and DOCK6 (blue) with an RMSD of 3.82Å, 8.03Å, and 6.74 Å from the native ligand pose (red), respectively. The lower-right compares the models by EDock with the composite weights (yellow), and two uniform weights of $w_{3} = 1$ (green, RMSD = 9.26 Å) and 0 (orange, RMSD 8.85 Å), respectively

In Fig. 9, we show an example which demonstrates the effect of the approach with variable van der Waals and pocket distance restraint weights. This is a target from the beta-hydroxydecanoyl thiol ester dehydrase complexed with unsaturated fatty acids (COACH ID: 1mkaA_BS02_DAC). The I-TASSER model has an acceptable global fold with TM-score = 0.853 but has severe atomic clashes with the native ligand in the pocket region. EDock has five different van der Waals weights, where only the model generated from the simulations with composite weight sets resulted in a correct ligand position with an RMSD = 3.82 Å, which compares favorably to the models from AutoDock Vina (8.03Å) and DOCK6 (6.74 Å).

#### Docking performance is more sensitive to the quality of local pocket than global fold

When analyzing the quality of protein structure models for docking, it is not enough to consider only metrics for overall protein topology such as TM-score [26]. Pocket structure quality is more relevant because docking conformations are sampled only near the pocket. To have a quantitative assessment of the local quality of the binding pocket, we define the binding pocket as the set of amino acid residues which have at least one heavy atom whose distance to the closest ligand heavy atom is below the sum of the van der Waals radii of the two involved atoms, plus a tolerance of 0.5 Å [28]. The pocket RMSD is calculated by directly superimposing the binding pockets of the receptor structures by the TM-score program. Additional file 1: Figure S10 shows the distribution of pocket RMSD for the DUDE and COACH datasets separately. It is shown that EDock was able to predict reasonable binding pocket structures, with a large portion of cases having a pocket RMSD < 2 Å, despite the variation in the global fold of the receptor structural models.

In Additional file 1: Table S8, we re-evaluate the performance of the docking methods on the targets whose binding-site distance error are < 8 Å and pocket RMSD is < 2 Å. Compared to the results in Table 3, the docking models by all three programs have better quality in Additional file 1: Table S8, due to the improved quality of the binding pocket, where the average pocket RMSD was reduced from 1.74 to 1.04 Å due to the filter. Similarly, the EDock models have lower RMSDs and center distances compared to the control methods, especially for the predicted structures of the COACH dataset. Here, the integration of the EDock binding site predictions helped to reduce the average ligand RMSD of AutoDock Vina from 10.41 (9.79) Å to 6.03 (6.86) Å and increase the number of cases with a center distance < 4 Å by 51 (15) in the COACH (DUDE) dataset, compared to the original AutoDock Vina without using binding site predictions. This data demonstrates again the usefulness of the COACH binding site predictions to guide blind docking experiments with low-resolution receptor structures.

**Fig. 10:**
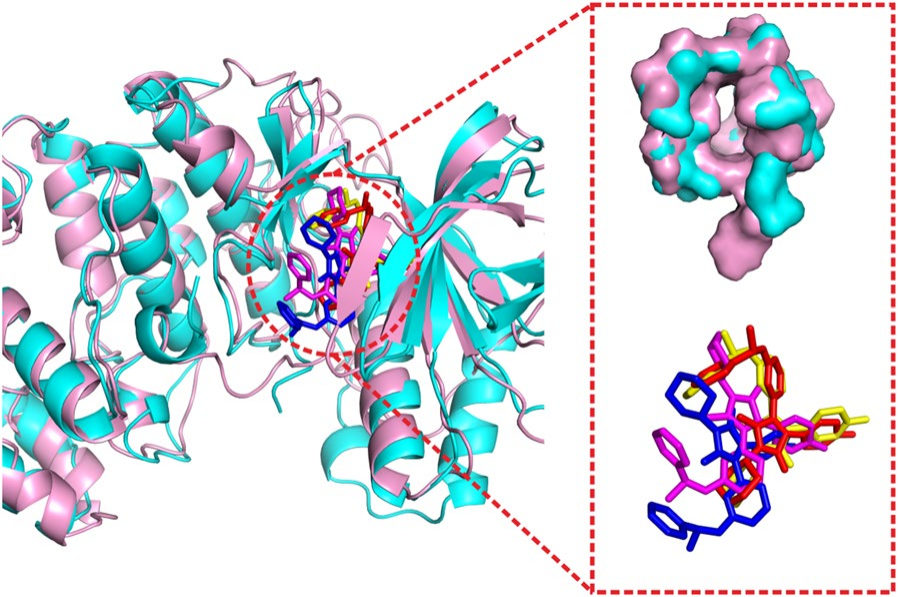
The docking performance on the P38 kinase domain bound with a Monocyclic Pyrazolone Inhibitor (COACH ID: 1ywrA_BS01_LI9). The I-TASSER receptor model has a relatively low TM-score (= 0.767, pink cartoon, left panel) but with a good binding cavity (pocket RMSD = 0.86 Å, upper-right panel). This results in a low ligand RMSD by EDock (1.40 Å, yellow), which compares favorably to AutoDock Vina (6.50 Å, magenta) and DOCK6 (7.82 Å, blue) as shown in the lower-left panel

In Fig. 10, we show an example from the P38 kinase domain in complex with a Monocyclic Pyrazolone Inhibitor (COACH ID: 1ywrA_BS01_LI9). The global receptor structure by I-TASSER has a relatively low TM-score (= 0.767) but a high-quality binding pocket with a pocket RMSD of 0.86 Å, which leads to EDock’s top ranked docking model possessing a ligand RMSD of 1.40 Å. The result indicates that while a high TM-score I-TASSER model is helpful to predict binding sites using COACH, the local pocket structure quality is more relevant to the final docking performance.

**Fig. 11:**
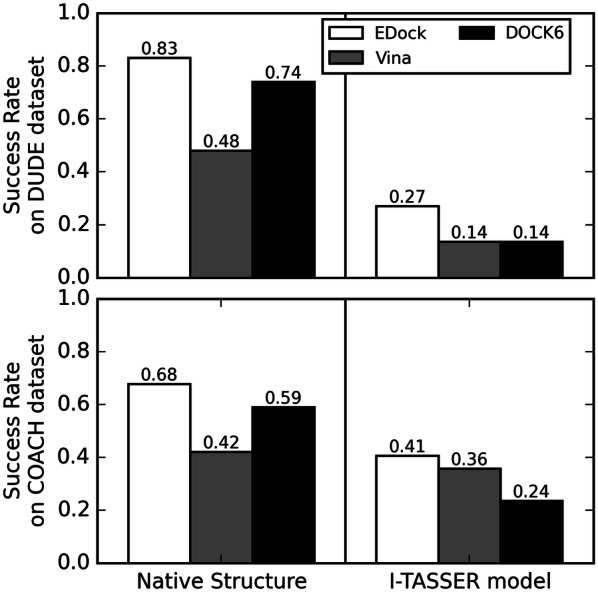
The success rates of different methods on DUDE and COACH datasets starting from predicted binding pockets. Here, a case is considered successful if the ligand RMSD is below 2 (or 5) Å with the receptor structure from experiment (or I-TASSER prediction)

Finally, we summarize in Fig. 11 the overall success rate of the three docking methods on the two datasets of DUDE and COACH with different receptor structure sources, where a docking result is considered successful if the ligand RMSD is below 2 (or 5) Å for cases when the native structure (or I-TASSER predicted model) is used. It is shown that all the docking programs achieved a higher success rate in the DUDE dataset when the experimental receptor structure is used (0.84, 0.47, and 0.75 versus 0.70, 0.44, and 0.60 by EDock, Vina and DOCK6, respectively), which is likely due to the relatively conserved binding pockets and rigid ligand structures with a relatively lower number of rotatable bonds in the dataset (Additional file 1: Figure S6). Somewhat unexpectedly, the success rates are all lower in the DUDE dataset than those in the COACH set if I-TASSER predicted models are used (0.41, 0.14 and 0.14 versus 0.59, 0.38 and 0.24 by EDock, Vina and DOCK6, respectively). This is, however, understandable considering the fact that the binding pockets in the COACH dataset have a lower pocket RMSD (= 0.98 Å) than those in the DUDE set (1.26 Å), even though the COACH dataset has a slightly lower TM-score (0.808) than the DUDE set (0.812). This data demonstrates again that the local quality of the binding pocket is more relevant to the final docking performance than the global topology of the receptor models.

### Flexible docking comparison

We also examine the ability of EDock flexible docking against other control methods, AutoDock Vina and DOCK6. In the REMC simulation, the intra-ligand van der Waals potential is added into the simulation energy function to evaluate ligand conformation changes. Random rotation of a single bond is implemented alongside rigid-body translations and rotations of the ligand molecule. Two ways of initializing the input ligand structures are used for benchmarking the EDock method, which both are applied to the protein–ligand pairs of the COACH dataset. One is that the crystal structure ligand pose is used for input. The other is when each single bond is randomly rotated to build a random ligand pose for input. We also compare the blind flexible docking performance based on holo-protein structures and I-TASSER predicted structures, which contain 315 and 180 targets, respectively, and whose binding sites are the same as with rigid body docking.

In Table 4, we present a summary of the flexible docking results of EDock compared with AutoDock Vina. For holo-protein structures, the average and median RMSD of EDock is slightly lower than AutoDock Vina for crystal and random ligand input. However, AutoDock Vina has a lower center distance between model and native than EDock. There are 159 (or 157) cases whose EDock RMSD were lower than Vina for crystal (or random) ligand input, respectively. For the predicted structures, EDock significantly outperforms Vina. The average and median RMSD of EDock is 4.85 Å (or 5.09 Å) for crystal (or random) ligand input, which is 1.39 Å (or 1.38 Å) lower than Vina. There are 118 (or 123) cases in which EDock was better than Vina for crystal (or random) ligand input, which corresponds to a *p*-value of 1.11E−06 and 7.75E−08 using a paired Student’s t-test, respectively.

**Table 4:** Summary of flexible docking results of EDock compared with Vina

Receptor structure	Input ligand structure	Method	RMSD (Å)		Center distance (Å)		Average RMSD < 2.0 (or 5.0 Å)
			Ave	Med	Ave	Med	
Holo-protein structure (315)	Crystal	EDock	*4.75*	*4.29*	2.44	1.59	*96*
		Vina	4.79	4.69	*2.14*	*1.55*	74
Holo-protein structure (315)	Random	EDock	*4.81*	*4.59*	2.51	1.83	*74*
		Vina	4.84	4.81	*2.17*	*1.55*	63
Predicted structure (180)	Crystal	EDock	*4.85*	*4.42*	*2.72*	*2.11*	*101*
		Vina	6.24	6.37	3.48	3.19	56
Predicted structure (180)	Random	EDock	*5.09*	*4.99*	*2.77*	*2.27*	*90*
		Vina	6.47	6.45	3.52	3.07	40

“Crystal” and “Random” represent the real ligand conformation and random rotatable conformation, respectively. The best performance is highlighted in italic font in each category

EDock is also compared with DOCK6, whose result is shown in Additional file 1: Table S9. There are 91 (and 162) cases out of 315 test targets for crystal (and random) ligand inputs for holo-protein structures, and 27 (and 70) out of 180 targets for predicted structures in which DOCK6 failed to produce a valid docking result. Additional file 1: Table S9 shows the flexible docking performance only for the targets for which DOCK6 produced a valid result. Although the results indicate that DOCK6 has better docking performance (RMSD = 4.49 and 4.23 Å) for crystal and random ligand input on holo-proteins, this is likely because the “anchor and grow” method of DOCK6 cannot obtain docking poses in many cases, an issue which is solved in EDock. However, when docking on predicted models, the average RMSD of EDock was 2.21 (and 2.59) Å lower than DOCK6 for for crystal (and random) ligand input, which results in 111 (and 73) targets for which EDock outperforms DOCK6.

#### The conserved rate of native binding sites for low-resolution structures

In the majority of this manuscript, we have compared the docking RMSD between the predicted pose and native pose to evaluate EDock, AutoDock Vina and DOCK6 for low-resolution structures. However, this metric may not be completely reasonable for evaluating the performance of docking on predicted models, as the binding site is usually somewhat warped, and the native pose is no longer reasonable as a “ground truth” (e.g. the native ligand pose sterically clashes with the residues of the binding pocket). Here we re-evaluate the number of conserved native binding contacts between ligand and protein for predicted structures. According to the docking pose on I-TASSER models, the binding site residues predicted to be in contact with the ligand can be obtained through determining the set of amino acid residues with an inter-atom distance to the closest ligand heavy atom below the sum of the van der Waals radii of the two involved atoms, plus a tolerance of 0.5 Å. Then, the predicted residues in contact with the ligand are compared with the native binding site. The precision $\left( {TP/\left( {TP + FP} \right)} \right)$, recall $\left( {TP/\left( {TP + FN} \right)} \right)$ and F1 score $\left( {2/\left( {1/precision + 1/recall} \right)} \right)$ are used to evaluate the consistency with native binding, where TP, FP and FN mean the number of true positive, false positive and false negative contacts relative to the native protein–ligand complex. In Additional file 1: Table S10, the F1 score and recall of EDock are 5.6% (or 13.6%) and 25.6% (or 30.8%) higher than AutoDock Vina (or DOCK6) for rigid body docking, while the precision is 8.6% (or 3.1%) lower than AutoDock Vina (or DOCK6). For flexible docking, we only compare with AutoDock Vina because of the cases failed by DOCK6. The F1 score and recall of EDock is 8.0% (5.5%) and 22.3% (20.4%) higher than AutoDock Vina, but the precision is 5.4% (6.8%) lower than AutoDock Vina for crystal (random) ligand input. This is because AutoDock Vina will dock the ligand in a site on the protein less amenable to binding than the native binding site in order to avoid the steric clashing. The fact that the resulting binding site is often less of a “pocket” than the original binding site makes the total number of binding contacts ($TP + FP$) smaller than native number ($TP + FN$), so the precision is higher than other methods. The comparison results in Additional file 1: Table S10 and Table 3 indicate EDock not only can predict more accurate docking positions but also can obtain more near native docking poses.

## Discussion

We developed a new method, EDock, for blind protein–ligand docking through replica-exchange Monte Carlo simulations. Starting from the structure of a protein receptor, the binding sites are first predicted through sequence-profile and local-structure based threading, where putative binding pockets are created by negative imaging of the predicted binding site. Next, a modified graph matching approach is extended to construct initial ligand poses, which are further refined by REMC simulations under a composite force field constrained by the binding site predictions and template ligand distance profiles.

The method was tested on two large-scale datasets from DUDE and COACH [10, 11], where EDock generated correct docking conformations with a ligand RMSD < 2 Å in 84% and 70% of the cases, respectively, based on the experimental receptor structures but using predicted binding sites. This compares favorably with widely used docking tools, including AutoDock Vina (with a success rate of 47% and 44%) and DOCK6 (75% and 60%), which start with the same binding site predictions. The results also showed the usefulness of profile-based binding site predictions, which can help improve other docking programs such as AutoDock Vina.

For the first time, the pipelines were examined based on low-resolution receptor structures as predicted by the state-of-the-art protein structure prediction method I-TASSER. As expected, the accuracy of the docking results is much lower than that based on experimental holo-structures due to the severe distortion of the binding pockets in the apo-models predicted by I-TASSER. Nevertheless, the number of acceptable cases with a ligand RMSD < 5 Å predicted by EDock is still higher than AutoDock Vina (by 67%) and DOCK6 (by 159%). One of the advantages in EDock is the use of REMC simulations, which can significantly refine the initial docking models created from graph matching. This was also manifested through the significant superiority of EDock over BSP-SLIM [23], another blind docking approach based only on negative imaging. Meanwhile, the REMC simulations allow for adoption of a composite weighting strategy in the parallel replica runs, which helps to accommodate different levels of local pocket distortion and steric overlaps between ligand and receptor atoms, as the latter often vary greatly from case to case. The data also revealed a higher sensitivity of the docking performance towards the local binding pocket quality than the global fold of the predicted receptor models. Although EDock’s average running time is slower than AutoDock Vina and DOCK6, (2.02 h compared 0.33 and 18.42 min, respectively), EDock can obtain diverse initial conformations by graph matching and select the most reasonable docking pose through simulation energy ranking. The decoys in the different local minimum energy landscape can be more thoroughly searched by REMC simulation relative to other programs. Finally, near native poses can be selected by XSCORE ranking for experimental receptor structure and SPICKER clustering for I-TASSER models. The SPICKER scoring system is superior to XSCORE for docking on predicted models and is not possible without the REMC simulation. Despite the promising success of EDock, docking ligands with low-resolution receptor structures remains a significant challenge. While the composite van der Waals weighting can help alleviate steric clashes, the atomic overlaps between ligand and receptor still exist and can impact the precise docking energy calculations. Thus, new flexible docking methods that can integrate both ligand and receptor structure changes will be essential to address this issue; related work is currently under development.

## Availability and requirements

Project Name: EDock.

Project home page: https://zhanglab.ccmb.med.umich.edu/EDock/.

Operating system: Binary in Linux, Source code platform independent.

Programming language: C++, Python.

Other requirements: GCC 7.4.0 or higher or compatible Linux operating system.

License: GNU GPL.

## Supplementary information

**Additional file 1: Figure S1**. Definition of solvent-exposed pocket grid points. **Figure S2**. The replica-exchange Monte Carlo protocol used in EDock. **Figure S3**. An illustrative example of REMC energy trajectories. **Figure S4**. The spherical coordinate system for generating randomly oriented rotation axis unit vectors. **Figure S5**. The conformation selection protocol extended from SPICKER. **Figure S6.** Distribution of ligand size and the number of rotatable bonds. **Figure S7**. Distribution of the number of pocket grid points. **Figure S8**. Distribution of the TM-scores of the receptor models predicted by I-TASSER. **Figure S9.** Comparison of ligand RMSD generated by different methods based on the I-TASSER predicted receptor models. **Figure S10.** The RMSD distribution of predicted binding pockets based on the I-TASSER structural models. **Table S1.** Summary of initial ligand docking conformations at different energy thresholds. **Table S2.** Summary of the parameters of the REMC simulations. **Table S3.** Parameters for the van der Waals energy potential. **Table S4.** Summary of the docking results of the top conformation on 180 I-TASSER predicted structures by different van der Waals weights. **Table S5.** Summary of docking performance at different box size and REMC swap number on 180 predicted structures targets of the COACH dataset. **Table S6.** Summary of the docking results of the top conformation on 391 experimental structures and 237 I-TASSER predicted structures by different ranking methods. **Table S7.** Summary of the blind docking result comparison between BSP-SLIM and EDock. **Table S8.** Summary of docking results on 160 targets that have receptor models from I-TASSER. **Table S9.** Summary of flexible docking results of EDock compared with DOCK6. **Table S10.** Summary of the conserved rate of native binding contacts of 180 predicted models for rigid and flexible docking.

## Data Availability

The datasets generated and analyzed during the current study as well as EDock source code are available at the EDock webserver, https://zhanglab.ccmb.med.umich.edu/EDock/.
