# Supplementary material for: EDock: blind protein–ligand docking by replica-exchange monte carlo simulation
Source: J Cheminform. 2020 May 27;12:37. doi: 10.1186/s13321-020-00440-9 (PMC7251717; doi:10.1186/s13321-020-00440-9)
Supplement: Supplementary file 1 — Additional file 1: Figure S1. Definition of solvent-exposed pocket grid points. Figure S2. The replica-exchange Monte Carlo protocol used in EDock. Figure S3. An illustrative example of REMC energy trajectories. Figure S4. The spherical coordinate system for generating randomly oriented rotation axis unit vectors. Figure S5. The conformation selection protocol extended from SPICKER. Figure S6. Distribution of ligand size and the number of rotatable bonds. Figure S7. Distribution of the number of pocket grid points. Figure S8. Distribution of the TM-scores of the receptor models predicted by I-TASSER. Figure S9. Comparison of ligand RMSD generated by different methods based on the I-TASSER predicted receptor models. Figure S10. The RMSD distribution of predicted binding pockets based on the I-TASSER structural models. Table S1. Summary of initial ligand docking conformations at different energy thresholds. Table S2. Summary of the parameters of the REMC simulations. Table S3. Parameters for the van der Waals energy potential. Table S4. Summary of the docking results of the top conformation on 180 I-TASSER predicted structures by different van der Waals weights. Table S5. Summary of docking performance at different box size and REMC swap number on 180 predicted structures targets of the COACH dataset. Table S6. Summary of the docking results of the top conformation on 391 experimental structures and 237 I-TASSER predicted structures by different ranking methods. Table S7. Summary of the blind docking result comparison between BSP-SLIM and EDock. Table S8. Summary of docking results on 160 targets that have receptor models from I-TASSER. Table S9. Summary of flexible docking results of EDock compared with DOCK6. Table S10. Summary of the conserved rate of native binding contacts of 180 predicted models for rigid and flexible docking. [file 13321_2020_440_MOESM1_ESM.docx]

**Additional file 1**

**Table of Contents**

**Supporting Figures**

- **Figure S1**. Definition of solvent-exposed pocket grid points.
- **Figure S2**. The replica-exchange Monte Carlo protocol used in EDock.
- **Figure S3**. An illustrative example of REMC energy trajectories.
- **Figure S4**. The spherical coordinate system for generating randomly oriented rotation axis unit vectors.
- **Figure S5**. The conformation selection protocol extended from SPICKER.
- **Figure S6.** Distribution of ligand size and the number of rotatable bonds.
- **Figure S7**. Distribution of the number of pocket grid points.
- **Figure S8**. Distribution of the TM-scores of the receptor models predicted by I-TASSER.
- **Figure S9.** Comparison of ligand RMSD generated by different methods based on the I-TASSER predicted receptor models.
- **Figure S10.** The RMSD distribution of predicted binding pockets based on the I-TASSER structural models.

**Supporting Tables**

- **Table S1.** Summary of initial ligand docking conformations at different energy thresholds.

# Table S2. Summary of the parameters of the REMC simulations.

- **Table S3.** Parameters for the van der Waals energy potential.
- **Table S4.** Summary of the docking results of the top conformation on 180 I-TASSER predicted structures by different van der Waals weights.
- **Table S5.** Summary of docking performance at different box size and REMC swap number on 180 predicted structures targets of the COACH dataset.
- **Table S6.** Summary of the docking results of the top conformation on 391 experimental structures and 237 I-TASSER predicted structures by different ranking methods.
- **Table S7.** Summary of the blind docking result comparison between BSP-SLIM and EDock.
- **Table S8.** Summary of docking results on 160 targets that have receptor models from I-TASSER.
- **Table S9.** Summary of flexible docking results of EDock compared with DOCK6.
- **Table S10.** Summary of the conserved rate of native binding contacts of 180 predicted models for rigid and flexible docking.

**References**

**Supporting Figures**


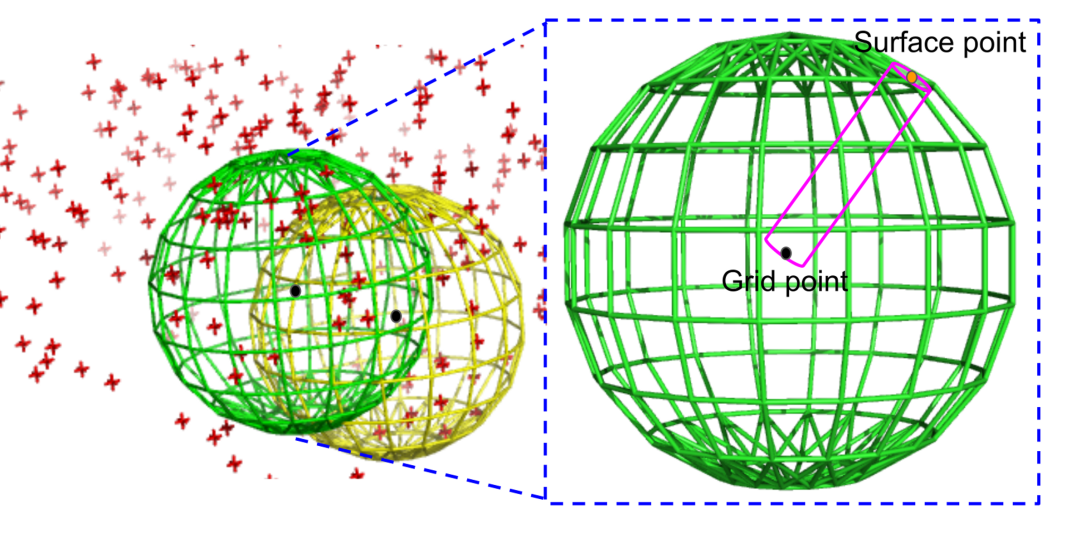


**Figure S1**. The definition of solvent-exposed pocket grid points. To judge if a grid point (represented by the black solid circles) is buried by the receptor atoms (red crosses), we draw a sphere centered at the grid point with a radius of 20 Å (green and yellow meshes). The surface of this sphere is split into 146 evenly distributed surface points (right inset). For each surface point (orange), a cylinder (magenta) is drawn whose radius is 2.0 Å and whose central axis is drawn from the grid point to this surface point. A grid point is defined as solvent-exposed, if less than half of the cylinders contain at least one receptor atom inside the cylinder space.


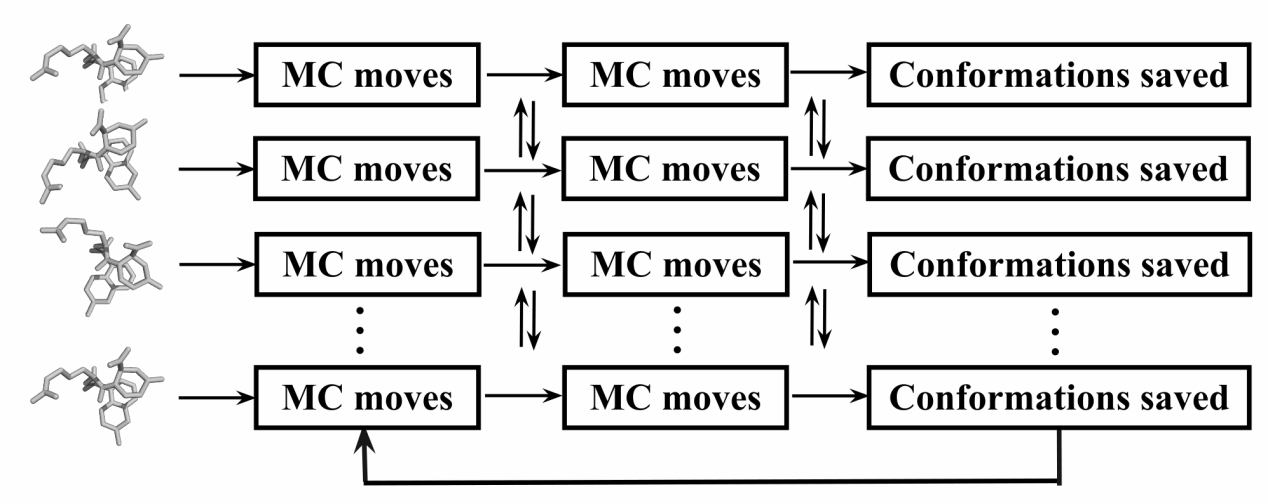


**Figure S2**. The replica-exchange Monte Carlo protocol used in EDock. A set of $N$ replicas of the docking simulation are performed in parallel [1], where swapping of the conformations between neighboring replicas is attempted periodically with acceptance based on the Metropolis criterion [2].


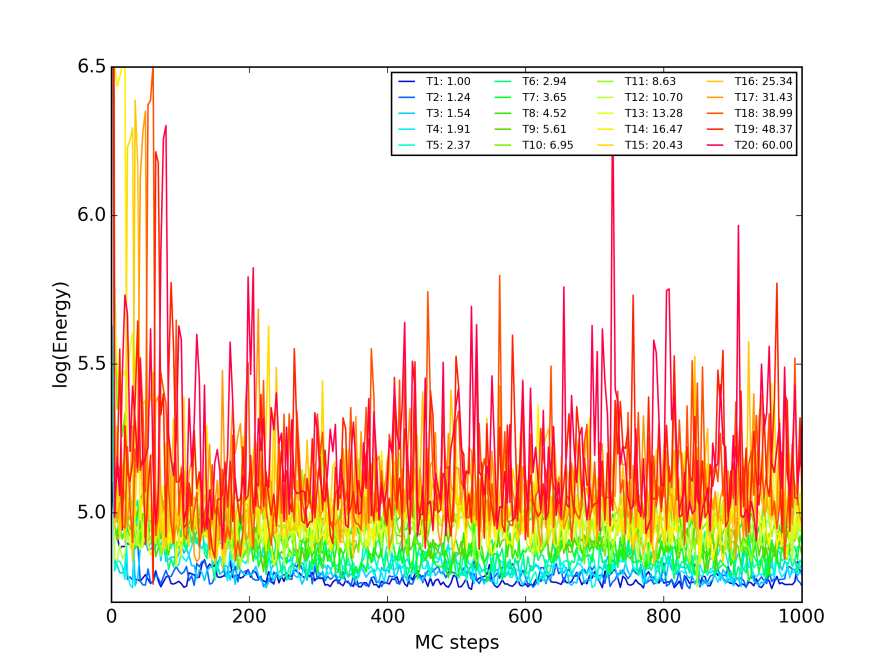


**Figure S3**. An illustrative example of REMC energy trajectories from 20 different replicas from rabbit phosphoglucose isomerase complexed with sorbitol-6-phosphate (COACH ID: 1xtbA_BS01_S6P).





**Figure S4**. The spherical coordinate system for generating randomly oriented unit rotation axis vectors.

**
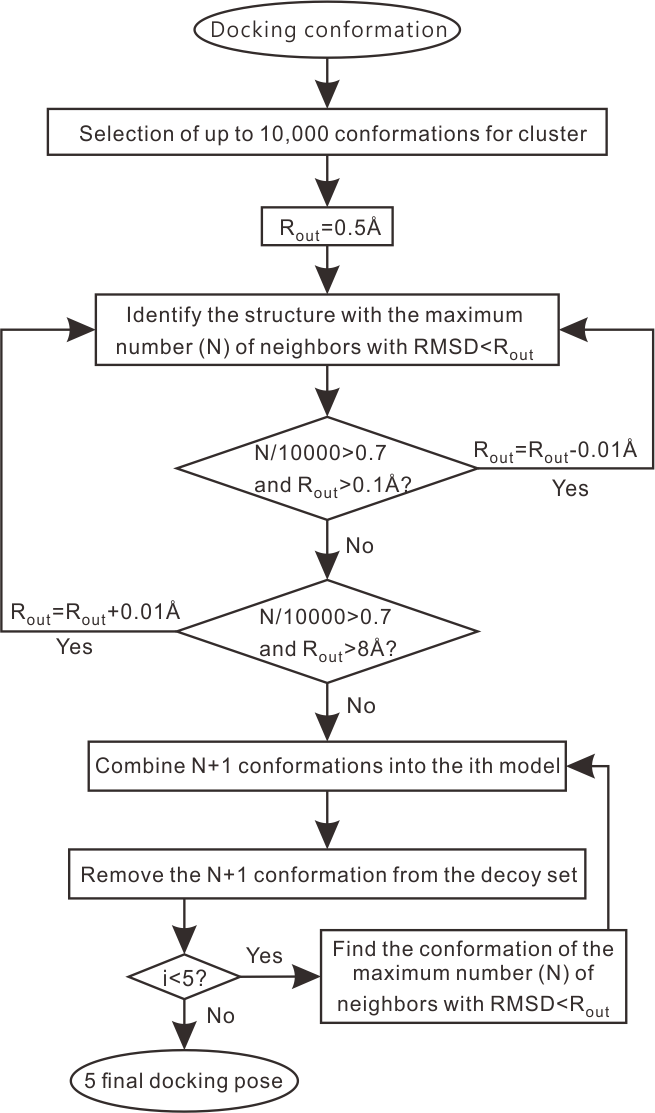
**

**Figure S5**. The conformation selection protocol extended from SPICKER clustering [3], where all cutoff parameters have been adjusted based on a training set.





**Figure S6.** The distribution of ligand size and number of rotatable bonds for the targets in the DUDE (upper panels) and COACH (lower panels) datasets.


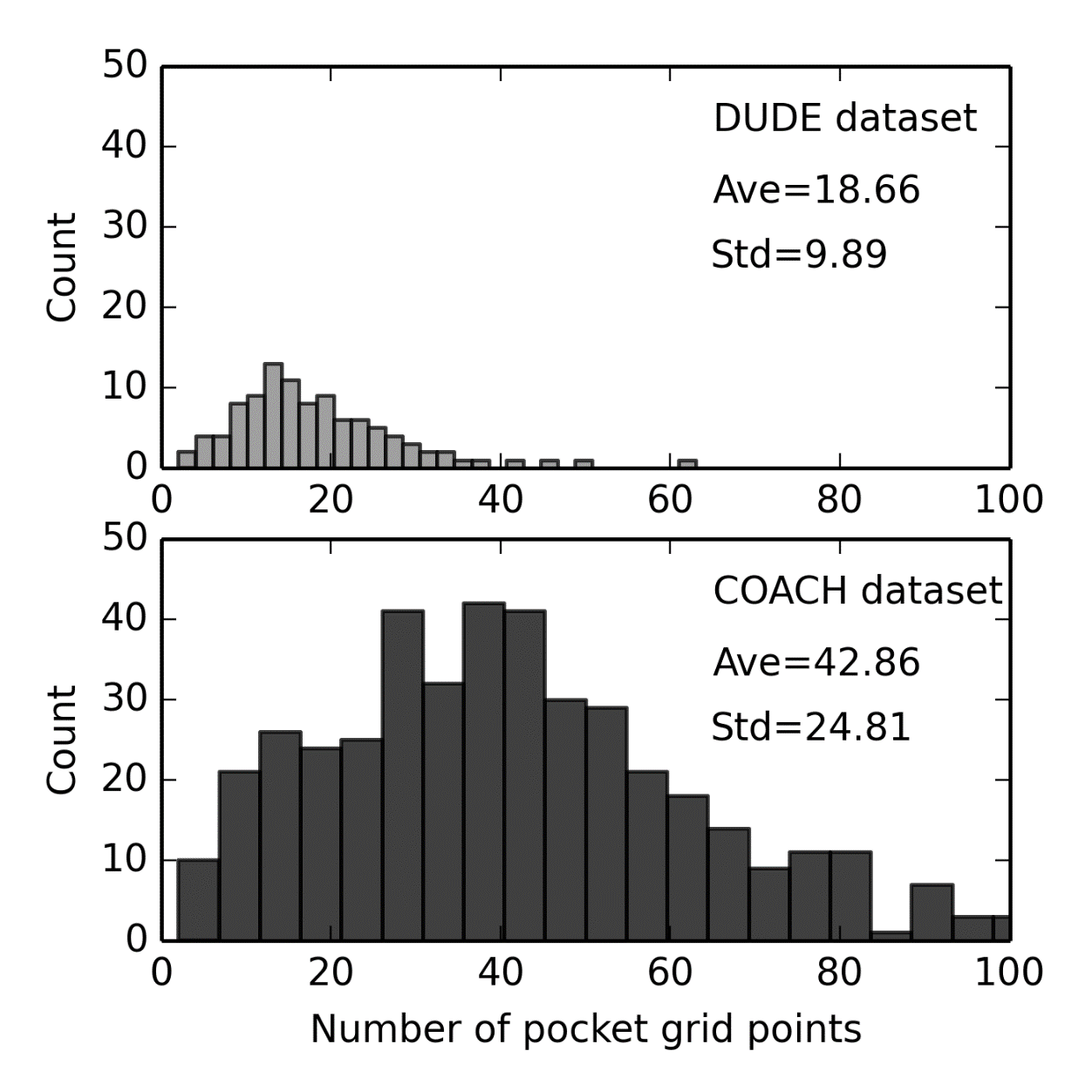


**Figure S7**. The distribution of the number of pocket grid points for the targets in the DUDE (upper panel) and COACH (lower panel) datasets.





**Figure S8**. The distribution of TM-scores of the receptor models predicted by I-TASSER after filtering out the close homologous templates for the DUDE dataset (left panel) and the COACH dataset (right panel).


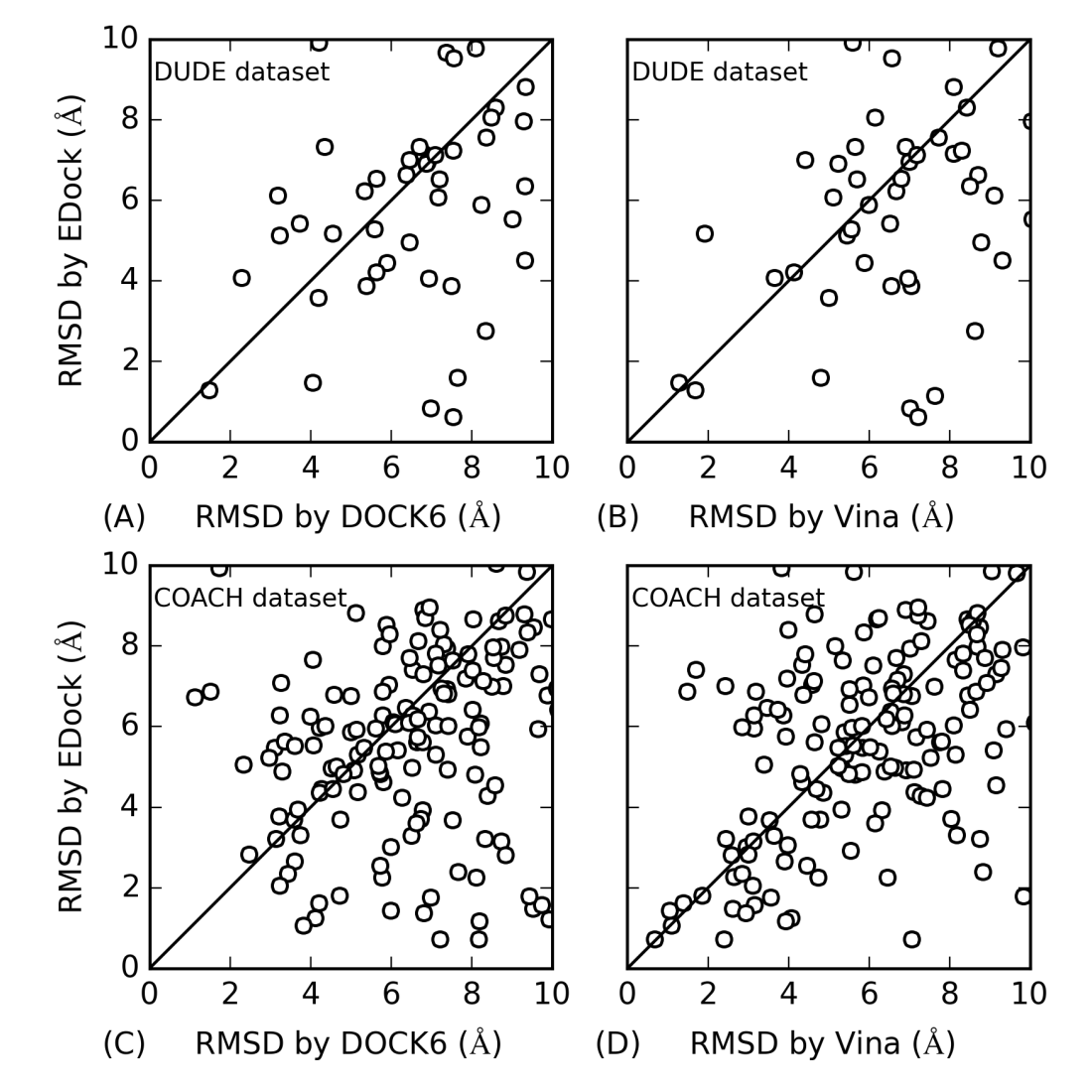


**Figure S9.** Comparison of ligand RMSD by different methods based on the I-TASSER predicted receptor models. (A, B) DUDE dataset; (C, D) COACH dataset.





**Figure S10.** The RMSD distribution of predicted binding pockets based on the I-TASSER structural models. (A) DUDE dataset (B) COACH dataset.

**Supporting Tables**

**Table S1** Summary of initial ligand docking conformations at different energy thresholds on 433 targets of the COACH dataset. “Threshold” is the maximum allowable energy value for the initial conformations. “RMSD” is the root-mean-square deviation between the native ligand and the graph matching conformation with the best XSCORE. “$n_{com}$” is the average number of initial conformations selected.

| Threshold (kcal/mol) | RMSD (Å) | $n_{com}$ |
| --- | --- | --- |
| 1E+4 | 3.67 | 114.63 |
| 1E+5 | 3.89 | 201.41 |
| **1E+6** | **3.86** | **295.43** |
| 1E+7 | 4.11 | 391.71 |

# Table S2. Summary of parameters of the REMC simulations with 20 replicas on the experimental receptor from the rabbit phosphoglucose isomerase complexed with sorbitol-6-phosphate (COACH ID: 1xtbA_BS01_S6P).

| $\boldsymbol{Index}$**^a^** | $\boldsymbol{T}_{\boldsymbol{i}}$**^b^** | $\boldsymbol{R}_{\boldsymbol{swap}}$**^c^** | $\boldsymbol{R}_{\boldsymbol{move}}$**^d^** | $\boldsymbol{\langle E\rangle}$**^e^** |
| --- | --- | --- | --- | --- |
| 1 | 1.00 | 0.28 | 0.078 | -39.50 |
| 2 | 1.24 | 0.63 | 0.127 | -37.66 |
| 3 | 1.54 | 0.64 | 0.168 | -35.73 |
| 4 | 1.91 | 0.73 | 0.226 | -33.19 |
| 5 | 2.37 | 0.69 | 0.286 | -31.88 |
| 6 | 2.94 | 0.77 | 0.333 | -29.50 |
| 7 | 3.64 | 0.76 | 0.383 | -27.34 |
| 8 | 4.52 | 0.74 | 0.446 | -24.19 |
| 9 | 5.61 | 0.82 | 0.484 | -21.10 |
| 10 | 6.95 | 0.89 | 0.516 | -18.54 |
| 11 | 8.63 | 0.94 | 0.525 | -16.85 |
| 12 | 10.70 | 0.92 | 0.537 | -15.54 |
| 13 | 13.28 | 0.90 | 0.548 | -13.71 |
| 14 | 16.47 | 0.88 | 0.549 | -10.59 |
| 15 | 20.43 | 0.89 | 0.581 | -8.81 |
| 16 | 25.34 | 0.91 | 0.593 | -5.39 |
| 17 | 31.43 | 0.90 | 0.613 | -2.52 |
| 18 | 38.99 | 0.92 | 0.613 | 1.40 |
| 19 | 48.37 | 0.94 | 0.616 | 7.82 |
| 20 | 60.00 | 0.47 | 0.620 | 13.75 |

# $\boldsymbol{Index}$: index of replicas

# $\mathbf{T}_{\mathbf{i}}$: temperature of *i*th replica

# $\boldsymbol{R}_{\boldsymbol{swap}}$: acceptance rate of global swaps

# $\boldsymbol{R}_{\boldsymbol{move}}$: acceptance rate of local Monte Carlo movements

# $\boldsymbol{\langle E\rangle}$: average energy for different replicas

**Table S3.** Parameters for the van der Waals energy potential.

| **Atom definition** | **Atom name** | **Radius**  **(**$\boldsymbol{r}_{\boldsymbol{i}}$**) (Å)** | **Well depth (**$\boldsymbol{\varepsilon}_{\boldsymbol{i}}$**) (kcal/mol)** |
| --- | --- | --- | --- |
| C | Carbon | 1.908 | 0.086 |
| C.3 | Carbon_All_sp3 | 1.908 | 0.1094 |
| H | Hydrogen | 0.6 | 0.0157 |
| H ( O ) | Hydrogen_hydroxyl | 0.1 | 0.001 |
| H ( S ) | Hydrogen_thiol | 0.6 | 0.0157 |
| H ( C.3 ) | Hydrogen_Aliphatic | 1.487 | 0.0157 |
| H ( C.3 ( O/N/S/F/Cl/Br ) ) | Hydrogen_Semipolar | 1.387 | 0.0157 |
| H ( C.3 ( 2 O/2 N/2 S/(N)(O)/(S)(O)/(S)(N)(2 F)/(2 Cl)/(2 Br)) ) | Hydrogen_2_Semipolar | 1.287 | 0.0157 |
| H ( C.3 ( N.4 ) ) | Hydrogen_1-3_Charged | 1.1 | 0.0157 |
| H ( C.ar / C.2) | Hydrogen_sp2 | 1.459 | 0.015 |
| H ( C.ar ( O /N) ) or H ( C.2 ( O /N) ) | Hydrogen_Ar_Sp | 1.409 | 0.015 |
| H ( C.ar ( 2 O/2 N/(N)(O) ) ) or H ( C.2 ( 2 O )/(2 N)/(N)(O) ) | Hydrogen_Ar_2_Sp | 1.359 | 0.015 |
| H ( C.1 ) | Hydrogen_alkyne | 1.459 | 0.015 |
| N | Nitrogen | 1.824 | 0.17 |
| O | Oxygen | 1.6612 | 0.21 |
| O.3 | Oxygen_ether | 1.6837 | 0.17 |
| O.3 ( H ) | Oxygen_alcohol | 1.721 | 0.2104 |
| O.3 ( 2 H ) | Oxygen_TIP3_water | 1.7683 | 0.152 |
| S | Sulfur | 2 | 0.25 |
| P | Phosphorus | 2.1 | 0.2 |
| F | Fluorine | 1.75 | 0.061 |
| Cl | Chlorine | 1.948 | 0.265 |
| Br | Bromine | 2.22 | 0.32 |
| Si | Silicon | 2.22 | 0.32 |
| I | Iodine | 2.35 | 0.4 |
| K | Potassium+ | 2.658 | 0.000328 |
| Na | Sodium+ | 1.868 | 0.00277 |
| Mg | Mg++ | 0.787 | 0.875 |
| Li | Li+ | 1.137 | 0.0183 |
| Rb | Rubidium | 2.956 | 0.00017 |
| Cs | Cesium | 3.395 | 0.0000806 |
| Ca | Calcium2+ | 1.326 | 0.4497 |
| Zn | Zinc | 1.1 | 0.0125 |
| Fe | Iron | 1.2 | 0.05 |
| Sr | Strontium++ | 1.742 | 0.118 |
| Ba | Barium++ | 2.124 | 0.047 |
| V | Vanadium | 2.1 | 0.32 |
| Du | Dummy | 0 | 0 |

**Table S4.** Summary of the docking results of the top conformation on 180 I-TASSER predicted structures using different van der Waals weights.

| Weight | Ligand RMSD (Å) | | Center distance (Å) | |
| --- | --- | --- | --- | --- |
|  | Ave | Med | Ave | Med |
| 0.001 | 6.47 | 3.51 | 6.43 | 3.05 |
| 0.004 | 6.31 | 3.43 | 6.57 | 2.98 |
| 0.005 | 6.19 | 3.43 | 6.25 | 3.08 |
| 0.01 | 6.15 | 3.40 | 6.26 | 2.88 |
| 0.02 | 6.14 | 3.32 | 6.28 | 2.88 |
| 0.04 | 6.63 | 3.58 | 6.63 | 3.12 |
| 0.05 | 6.45 | 3.40 | 6.54 | 2.95 |
| 0.1 | 6.56 | 3.71 | 6.44 | 3.27 |
| 0.5 | 6.74 | 3.94 | 6.81 | 3.56 |
| 1 | 6.85 | 4.02 | 6.88 | 3.71 |
| [0.001,0.004] | 6.49 | 6.65 | 6.65 | 3.10 |
| [0.001,0.004,0.02] | 6.09 | 3.39 | 6.34 | 3.06 |
| [0.001,0.004,0.02,0.01] | 6.16 | 3.45 | 6.31 | 3.09 |
| [0.001,0.004,0.02,0.01,1] | 6.09 | 3.45 | 6.19 | 3.06 |

**Table S5****.** Summary of docking performance at different box size and REMC swap number on 180 predicted structures targets of the COACH dataset. The pose with the highest XSCORE from 40 initial conformations by graph matching is compared for evaluating the box size parameter. The pose with highest XSCORE of the docking decoys by EDock, Vina, and DOCK6 is compared for evaluating the swap number in the REMC simulation. “Average Number of grid points” means the average number of pocket grid points generated in binding pocket construction.

| Parameter | value | Average Number of grid points | RMSD (Å) | | Center distance (Å) | |
| --- | --- | --- | --- | --- | --- | --- |
|  |  |  | Ave | Med | Ave | Med |
| Box size | 10 | 8.18 | 10.10 | 9.64 | 7.89 | 7.03 |
|  | **20** | **26.25** | **7.56** | **7.33** | **4.97** | **4.18** |
|  | 30 | 66.99 | 10.88 | 10.26 | 8.76 | 7.99 |
| REMC  swap number | 100 | 26.25 | 7.19 | 7.03 | 4.33 | 3.93 |
|  | **200** | **26.25** | **7.10** | **6.85** | **4.40** | **3.99** |
|  | 400 | 26.25 | 7.21 | 7.23 | 4.42 | 3.91 |

**Table S6.** Summary of the docking results of the top conformation on 391 experimental structures and 237 I-TASSER predicted structures by simulation energy ranking, XSCORE ranking and SPICKER clustering.

| Dataset | Ranking methods | RMSD (Å) | | Center distance (Å) | |
| --- | --- | --- | --- | --- | --- |
|  |  | Ave | Med | Ave | Med |
| Experimental  (391) | Simulation energy | 2.46 | 0.47 | 1.53 | 0.37 |
|  | **XSCORE** | **2.03** | **0.40** | **1.15** | **0.30** |
|  | SPICKER clustering | 3.22 | 0.83 | 2.03 | 0.60 |
| Predicted  (237) | Simulation energy | 5.01 | **4.63** | 3.04 | 2.41 |
|  | XSCORE | 5.53 | 5.58 | 3.01 | 2.55 |
|  | **SPICKER clustering** | **4.82** | 4.83 | **2.76** | **2.33** |

**Table S7.** Summary of the blind docking result comparison between BSP-SLIM and EDock on 248 targets for which BSP-SLIM could generate a final model. The protein-ligand pairs are merged from both the DUDE and COACH datasets. Receptor structures are either from experimental solution (upper panel) or from I-TASSER prediction. Results with the best performance are highlighted in bold font.

| Receptor structures | Method | RMSD (Å) | | Center distance (Å) | |
| --- | --- | --- | --- | --- | --- |
|  |  | Ave | Med | Ave | Med |
| Experimental  (248) | BSP-SLIM | 8.02 | 6.36 | 5.80 | 2.43 |
|  | **EDock** | **6.49** | **1.16** | **5.52** | **0.70** |
| I-TASSER  (248) | BSP-SLIM | 10.02 | 7.91 | 7.42 | 4.02 |
|  | **EDock** | **8.72** | **6.74** | **6.82** | **3.88** |

**Table S8.** Summary of the docking results on 160 targets that have receptor models from I-TASSER with a binding site error < 8 Å and a pocket error <2 Å. ‘Ave’ and ‘Med’ represent the average and median values, respectively. The best performance is highlighted in bold font in each category.

| Dataset | Method | RMSD (Å) | | Center distance (Å) | | Average RMSD < 5 Å |
| --- | --- | --- | --- | --- | --- | --- |
|  |  | Ave | Med | Ave | Med |  |
| DUDE  (37) | **EDock** | **5.41** | **5.15** | **2.92** | **2.83** | **15** |
|  | DOCK6 | 7.08 | 7.09 | 3.91 | 3.36 | 5 |
|  | Vina | 6.86 | 6.90 | 3.34 | 2.90 | 5 |
| COACH  (123) | **EDock** | **4.47** | **3.78** | **2.58** | **1.90** | **73** |
|  | DOCK6 | 7.03 | 6.99 | 4.43 | 4.21 | 29 |
|  | Vina | 6.03 | 5.98 | 3.41 | 3.05 | 47 |

**Table S9.** Summary of flexible docking results of EDock compared with DOCK6. ‘Crystal’ and ‘Random’ represent the real ligand conformation and random conformation as input, respectively.

| Receptor structure | Input ligand structure | Method | RMSD (Å) | | Center distance (Å) | | Average RMSD <2.0 (5.0 Å) |
| --- | --- | --- | --- | --- | --- | --- | --- |
|  |  |  | Ave | Med | Ave | Med |  |
| Holo-protein structure  (224) | Crystal | EDock | 4.74 | 4.22 | 2.43 | 1.77 | 65 |
|  |  | Vina | 4.75 | 4.45 | 2.08 | 1.61 | 55 |
|  |  | DOCK6 | **4.49** | **3.85** | **2.00** | **1.23** | **70** |
| Holo-protein structure  (153) | Random | EDock | 4.87 | 4.69 | 2.41 | 1.83 | 33 |
|  |  | Vina | 4.78 | 5.00 | 2.11 | 1.65 | 31 |
|  |  | DOCK6 | **4.23** | **3.51** | **2.02** | **1.34** | **56** |
| Predicted structure  (153) | Crystal | EDock | **4.87** | **4.40** | **2.73** | **2.05** | **86** |
|  |  | Vina | 6.30 | 6.54 | 3.54 | 3.25 | 48 |
|  |  | DOCK6 | 6.99 | 6.79 | 4.21 | 4.73 | 32 |
| Predicted structure  (110) | Random | EDock | **5.03** | **4.95** | **2.72** | **2.03** | **56** |
|  |  | Vina | 6.62 | 6.49 | 3.64 | 3.16 | 23 |
|  |  | DOCK6 | 7.62 | 6.87 | 4.91 | 3.57 | 26 |

**Table S10.** Summary of the conserved rate of native binding contacts of 180 predicted models for rigid and flexible docking.

| Docking | Input ligand structure | Method | Precision | Recall | F1 |
| --- | --- | --- | --- | --- | --- |
| Rigid | Crystal | EDock | 0.564 | **0.749** | **0.632** |
|  |  | Vina | **0.650** | 0.534 | 0.576 |
|  |  | DOCK6 | 0.595 | 0.441 | 0.496 |
| Flexible | Crystal | EDock | 0.599 | **0.766** | **0.658** |
|  |  | Vina | **0.653** | 0.534 | 0.578 |
| Flexible | Random | EDock | 0.589 | **0.760** | **0.649** |
|  |  | Vina | **0.657** | 0.556 | 0.594 |

**References**

1. R. H. Swendsen and J. S. Wang (1986) Replica Monte Carlo simulation of spin glasses. Physical Review Letters 57:2607-2609.

2. N. Metropolis, A. W. Rosenbluth, M. N. Rosenbluth, A. H. Teller and E. Teller (1953) Equation of state calculations by fast computing machines. J Chem Phys 21:1087-1092.

3. Y. Zhang and J. Skolnick (2004) SPICKER: A clustering approach to identify near-native protein folds. J Comput Chem 25:865-871.
